# Demographic History, Population Structure, and Local Adaptation in Alpine Populations of *Cardamine impatiens* and *Cardamine resedifolia*

**DOI:** 10.1371/journal.pone.0125199

**Published:** 2015-05-01

**Authors:** Lino Ometto, Mingai Li, Luisa Bresadola, Enrico Barbaro, Markus Neteler, Claudio Varotto

**Affiliations:** Department of Biodiversity and Molecular Ecology, Research and Innovation Centre, Fondazione Edmund Mach, Via E. Mach 1, 38010 San Michele all′Adige (TN), Italy; CNR, ITALY

## Abstract

Species evolution depends on numerous and distinct forces, including demography and natural selection. For example, local adaptation and population structure affect the evolutionary history of species living along environmental clines. This is particularly relevant in plants, which are often characterized by limited dispersal ability and the need to respond to abiotic and biotic stress factors specific to the local environment. Here we study the demographic history and the possible existence of local adaptation in two related species of Brassicaceae, *Cardamine impatiens* and *Cardamine resedifolia*, which occupy separate habitats along the elevation gradient. Previous genome-wide analyses revealed the occurrence of distinct selective pressures in the two species, with genes involved in cold response evolving particularly fast in *C*. *resedifolia*. In this study we surveyed patterns of molecular evolution and genetic variability in a set of 19 genes, including neutral and candidate genes involved in cold response, across 10 populations each of *C*. *resedifolia* and *C*. *impatiens* from the Italian Alps (Trentino). We inferred the population structure and demographic history of the two species, and tested the occurrence of signatures of local adaptation in these genes. The results indicate that, despite a slightly higher population differentiation in *C*. *resedifolia* than in *C*. *impatiens*, both species are only weakly structured and that populations sampled at high altitude experience less gene flow than low-altitude ones. None of the genes showed signatures of positive selection, suggesting that they do not seem to play relevant roles in the current evolutionary processes of adaptation to alpine environments of these species.

## Introduction

Changes in population size and population subdivision are common features of most species′ evolutionary history. Depending on the geographic distribution of the populations, species may consist of more or less isolated populations, where neutral and non-neutral forces may have distinct effects. These forces, combined with the degree of inter-population migration and the local effective population size, define the relative role of genetic drift and selection in shaping both intra- and inter-population genetic differentiation and population structure [1–4]. The evolutionary history of a species can thus be seen as the combined result of global and local phenomena, and is determined by a balance between adaptation and population demographic history.

Population structure typically results from a combination of demographic (e.g., founder effects, bottlenecks, dispersal) and adaptive factors (e.g., differential selection along biotic and abiotic environmental gradients). Distinguishing the effects of such factors at the genetic level can be rather challenging. One approach relies on disentangling the effects of natural selection, which act at the locus-specific level, from the genome-wide effects of demographic processes. For instance, it is possible to compare the degree and pattern of genetic differentiation (e.g., *F*_ST_, allele frequencies) of candidate and putatively neutral loci along the geographic or environmental gradient of interest [5–9]. This approach gains considerably more power when combined with the inference of the demographic history and the structure of the population of interest, which help define the neutral model basis upon which testing single locus patterns of evolution [10–14].

Plants are an ideal system to investigate the occurrence and relevance of local adaptation, since, compared to most animals, their evolution is highly bound to their sessile nature and relatively limited dispersal capacity. Association mapping studies and reciprocal transplantation experiments have provided extensive evidence for intraspecific genetic variation and local adaptation for many traits [15–20]. One of the strongest selective forces affecting plants evolution and distribution is certainly climate [21]. Plants′ distributional ranges often encompass climatic gradients with strong fitness effects, as day length, water availability, and temperature, which impose locality-restricted selective pressures. Therefore, genes involved in the response to climatic gradients may harbor distinct functional alleles associated with the local conditions. For example, in *Arabidopsis thaliana*, polymorphism patterns associated with climatic gradients have been interpreted as signatures of local adaptation to sodium accumulation [22], cold response [23,24], and flowering time [25]. Likewise in *Populus balsamifera* there is evidence for adaptive variation along a latitudinal gradient in genes involved in phenology [26]. The results of these studies not only provided valuable information on the nature, speed, and mode of adaptive evolution in plants, but also offer the opportunity to understand the adaptive potential to ecological and environmental changes, including climate change [27,28].

In this study we estimated the demographic history and population structure of two congeneric Brassicaceae species that occupy separate habitats along the elevation gradient, and used this information to test the occurrence of local adaptation to temperature and water availability. The first of the two species, *Cardamine resedifolia*, is a perennial species usually growing in siliceous substrates in open spaces between 1,500 and 3,500 meters above sea level [29], and occasionally found in rocky grounds within coniferous forests (LO, personal observation). In contrast, *Cardamine impatiens* is a nemoral species usually found in deciduous or coniferous forests between 300 and 1,500 meters above sea level [29]. The populations analyzed in this study were sampled in the Italian Alps, whose orography (deep valleys and high mountains) represents a strong determinant of both species′ distribution and, by affecting gene flow, can result in population structure. Ice ages may have further shaped the demographic history of Alpine populations during the Quaternary [30]. In addition, the environmental and ecological factors associated with the disjointed altitudinal ranges evidently impose distinct selective pressures to the two species. For instance, the analysis of the patterns of synonymous and non-synonymous substitutions in *C*. *resedifolia* revealed rapid evolution of genes involved in cold response and slow evolution of genes involved in photosynthesis [31]. Conversely, in *C*. *impatiens* rates of evolution in genes involved in responses to cold and osmotic stresses and in photosynthesis were similar to those of the other genes, suggesting either weaker selective pressures or less efficient positive selection in this species [31]. Furthermore, several genes were detected as putative targets of positive selection in either of the two species, including genes that are annotated as involved in the response to salt stress and drought in *A*. *thaliana* [31]. Given that temperature and rainfall precipitation vary along their distributional range, the results also suggest that the two species may have undergone local adaptation to such climatic conditions.

We studied patterns of genetic variability at 19 genes in 60 individuals each of *C*. *resedifolia* and *C*. *impatiens* sampled from populations close to either the upper or the lower limits of the species′ distributional range in the Italian Alps (Trentino). All genes were readily sequenced by 454 pyrosequencing and included five outliers identified by Ometto *et al*. [31] as putative targets of positive selection in either of the two *Cardamine* species.

## Materials and Methods

### Samples

The populations used in this study were chosen to cover the species′ distribution range across Trentino (Italy). For both species, we sampled 6 individuals in each of 10 populations (S1 Table). For *C*. *impatiens* we sampled five populations located in the upper margin of the species′ range (henceforth H-populations; range 1,247–1,557 m; S1 Table) and five populations located in the lower margin of the species′ range (henceforth L-populations; range 337–749 m; S1 Table). Similarly for *C*. *resedifolia* we sampled five L-populations at an altitudine between 1,245–1,638 m and five H-populations between 2,129–2,526 m (S1 Table). Attention was paid to collect sufficiently spaced individuals to maximize genetic diversity in our samples. Upon collection, plants were stored in separate bags and either immediately dried with silica gel or preserved at low temperature until freezing at -80°C in the laboratory. Dried/frozen tissue was finely ground in liquid nitrogen using a ceramic pestle, and DNA extracted using CTAB buffer [32,33]. Quantification and quality of the DNA was assessed on 1% agarose gels.

### Gene selection and PCR reactions

We employed a high-throughput approach in which tagged PCR products for each gene and individual (i.e. amplicons) were sequenced at once using the Roche 454 GS-FLX sequencing technology. We sequenced 19 genes, including: (i) four candidate genes for the adaptation to high altitude [31]; (ii) seven genes annotated in *Arabidopsis thaliana* as involved in stress-responses typical of high altitude habitats; and (iii) eight putatively neutral genes randomly chosen among the *C*. *resedifolia* and *C*. *impatiens* orthologs that did not display any signature of positive selection in Ometto *et al*. [31] (see S2 Table for an overview). Primer design was based on the conservation of the orthologous *Cardamine* sequences sequenced by Ometto *et al*. [31]. These genes were aligned to full-length *A*. *thaliana* orthologues to predict intron/exon boundaries and evaluate putative gene lengths. Primers were then designed to delimit partially overlapping sequences with length compatible with 454 read size (~450 base pairs, bp). A list of primers and PCR conditions used is provided in S3 Table.

### Amplicon sequencing with Roche 454

The PCR products of each sample were pooled in a short (amplicon size <600 bp) and a long pool (>600 bp). Each of the resulting pools was purified with Agencourt AMPure XP kit (Beckman Coulter), blunt ended, methylated with CpG methyltransferase, and ligated to one of 120 sample-specific custom barcoded adapters (MIDs). Following the quantification with the Quant-iT Picogreen dsDNA Assay kit (Invitrogen) in a Synergy 2 fluorometer (BioTek), pools were normalized and combined into a short and a long super-pool to maximize sequencing efficiency. The two super-pools were then dephosphorylated and digested with the restriction enzyme *Srf*I (New England Biolabs) according to the manufacturer′s instructions. To remove residual enzymatic activity, all steps were followed by purification with SPRI beads (Beckman Coulter). Library preparation for 454 sequencing was carried out using a modified GS FLX Titanium Rapid Library preparation kit. In particular, the A-tailing of the two super-pools was performed in a total volume of 20 μl containing 200 ng of DNA, 1 x RL buffer, 2 mM dATP, and 5 unit of Taq polymerase (Sigma) at 72°C for 20 min; subsequently, 2.5 μl of ATP from the same kit was added for adapter ligation. Library quantitation was performed with the Kapa Library Quantification kit (Resnova). EmPCR library was performed according to the GS FLX Titanium emPCR Method Manual—Lib-L LV protocol for the short super-pool, and according to the Long Fragment Lib-A emPCR Amplification protocol for the long super-pool library. Finally, the two libraries were sequenced separately in a two-region picotiter plate of a GS FLX Titanium instrument (Roche 454 Life Science) using GS FLX Titanium Sequencing kit (Roche) according to the manufacturer′s instructions.

### Assembly

We used the *GS Amplicon Variant Analyzer* (AVA) application version 2.3 (Roche) to assign each read to the correct individual/gene assembly. Specifically, AVA associated a read to the appropriate individual based on its MID, and to the appropriate gene based on its match to the *A*. *thaliana* reference sequence. This process assigned 460,580 reads to one of the 2,276 assemblies (individual x gene combination). Assembled reads had mean length ± standard deviation of 395.0 ± 110.6 bp. To improve the alignments, we imported the assemblies and the relevant reference sequences into Geneious v5.3 [34], and re-assembled the reads to the reference with the “fine-tuning” parameter set to maximum. Depth of coverage of each assembly was then calculated using a custom perl script by counting the number of reads covering each position in the assembly.

### Consensus building and polymorphism detection

Identification of heterozygous sites, characterization of the alleles, and haplotype reconstruction were done using a custom perl pipeline. The identification of heterozygous sites is often complicated by the possible presence of sequencing errors [35], or errors in the PCR and alignment phases. Since the correct sequence of our genes/individuals was unknown, we indirectly estimated the error rate by analyzing plastidial sequences generated during the same 454 run [36]. Because chloroplasts are haploid, intra-accession polymorphisms are virtually absent and, therefore, mismatches result exclusively from sequencing errors. We evaluated the probability of calling a false allele in our assembly as a function of the number *n* of reads covering the polymorphic site, the number *k* of reads with the minor allele, and the error rate *f*_M_. This probability follows a binomial distribution and can be written as

$$P_{\text{false}}\text{=}P\left(k\text{;}n\text{, }f_{\text{M}}\right)\text{=}\left(\begin{array}{c} n\\ k \end{array}\right)f_{\text{M}}{}^{k}{\left(\text{1-}f_{\text{M}}\right)}^{n-k}$$

The variable *f*_M_ corresponds to the mean error rate estimated across all regions and polymorphism types described in Fior *et al*. [36], namely *f*_M_ = 0.024. We then iteratively calculated *P*_false_ for increasing values of *n*, whilst setting *k* equal to the smallest integer following 0.3 ×*n*. We estimated that a minimum depth of coverage of *n* = 5 gives a reasonably low probability of a false polymorphism detection of *P*_false_ = 0.0055. Subsequently, we scanned each assembly for the presence of polymorphic sites covered by at least 5 reads and with the minor allele present in more than 30% of them. To facilitate haplotype reconstruction, we also included sites with insertion/deletion polymorphisms. The script eventually recorded the two alleles, the read they were found in, and the relative position within the assembly. This information was used to reconstruct minimal haplotypes for each read of the assembly. Since minimal haplotypes overlapped only partially, we identified the complete region haplotypes by visually recognizing consistent association between consecutive alleles. We identified polymorphisms in a total of 222 individual/gene combinations (9.8% of the total), 93 of which in *C*. *resedifolia* and 125 in *C*. *impatiens*. In 14 assemblies, haplotype phasing was hindered by the presence of a pair of consecutive polymorphic sites not covered by the same reads. We therefore randomly picked one partial haplotype from the two halves marked by the discontinuity and built two putatively complete minimal haplotypes. This reconstruction does not affect measures of gene polymorphism and divergence, nor the population structure and association analyses. Following the minimal haplotype identification, we used Geneious to build the consensus sequence of each assembly by retaining the most common nucleotide observed across reads. Finally, we incorporated the minimal haplotype(s) into the consensus by using the known alleles′ positions, eventually generating two sequences per gene for each individual. All sequences have been deposited in GenBank under the accession numbers KJ427834–KJ432279.

We used the MUSCLE algorithm [37] implemented in Geneious to align all sequences from each gene. The *A*. *thaliana* orthologous sequence was added to the alignment to allow the identification of intron/exon boundaries in the *Cardamine* sequences and trim incomplete codons at the 5′ and 3′ ends of the alignment.

### Population genetic structure

We used the model-based clustering method implemented in the software STRUCTURE [38,39] to infer the genetic structure of the *C*. *impatiens* and *C*. *resedifolia* populations. For each species we used as input data a matrix containing all intraspecific variable sites (excluding singletons). We ran the program using the admixture model with uncorrelated allele frequencies and no location information (LOCPRIOR = 0). We run 10 independent replicates with a number *K* of clusters ranging from 1 to 6, with a burn-in period of 200,000 iterations followed by another 1,000,000 iterations. The putative optimal *K* was calculated by identifying the value at which the likelihood of the data reaches a plateau and using the approach of Evanno *et al*. [40]. Individual membership coefficients for each cluster were plotted using the DISTRUCT program [41].

In a second approach, the inference of population structure was based on the explicit use of both genetic and spatial information. We used Geneland [42] to simultaneously estimate the number of clusters and their geographical distribution in space. Because spatial information was available only for the whole population, we set the parameter delta.coord = 10^-6^ to account for the uncertainty of the spatial coordinates of individuals. For each species we run five (ten for *C*. *impatiens*) replicate analyses, each with a burn-in of 200,000 iterations followed by another 10,000,000 iterations and a thinning of 100. Analyses were done using the uncorrelated frequency model, and the number *K* of clusters ranged between 1 and 12.

Finally, we verified whether geographically restricted gene flow generated genetic structure by testing for isolation by distance in each species. Specifically, the correlation between the matrix of pairwise *F*_ST_ between populations (estimated by DnaSP [43]) and the matrix of geographic distances (calculated using the coordinates given in S1 Table) was tested by a Mantel test as implemented in the IBDWS application [44]. Tests were performed using the raw, as well as the ln-transformed data. We also estimated the actual differentiation index *D*_est_ proposed by Jost [45] using the web-based program SMOGD [46]. For each pairwise comparison, we calculated the between genes average of the across-alleles harmonic means.

### Demographic inference

**Fig 1:**
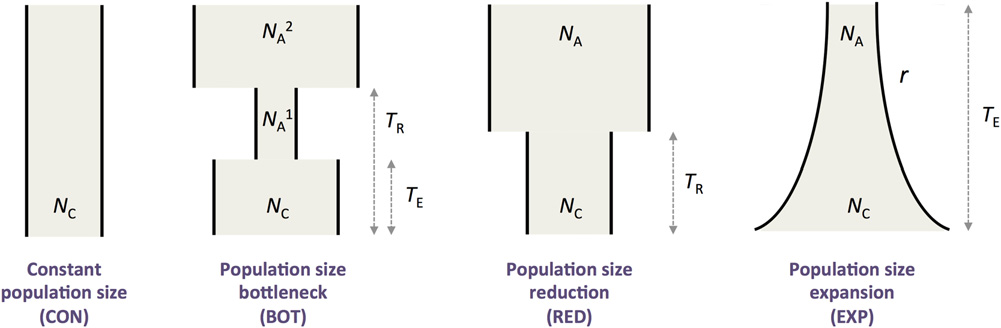
Demographic models investigated by Approximate Bayesian Computation (ABC). The four demographic scenarios are described by 1–5 parameters. *N*_C_ = Current population size; *N*_A_ = Ancestral population size; *T*_R_ = Time of population size reduction; *T*_E_ = Time of population size expansion; *r* = exponential growth parameter ($N_{\text{C}}\;=\;N_{\text{A}}\times e^{rT_{\text{E}}}$).

We used the Approximate Computational Bayesian (ABC) framework implemented in the package ABCtoolbox [47] to infer the putative demographic history of the sampled populations of *C*. *impatiens* and *C*. *resedifolia*. For both species, we simulated four simple demographic history scenarios (Fig 1): constant population size (CON), population size bottleneck (BOT), population size expansion (EXP), and population size reduction (RED).

The four models were described by one to five parameters, including population size and time of population size changes, to which we added priors for the mutation rate (1×10^-9^–5×10^-7^ mutations/site/generation) and the recombination rate (0–1×10^-8^ recombination events/site/generation). As summary statistics we used the number of segregating sites, *S*, and the average pairwise difference per base pair, π [48]. Overall we simulated a total of 1,000,000 sets of demographic parameters for each demographic model and species. To account for variation across loci, each gene was simulated independently using the same demographic scenario parameters, and we subsequently estimated mean and standard deviation across genes. Since coding regions may be subject to selective pressures, we only considered the polymorphism pattern of intronic regions, which were available for 15 genes (Tables 1 and 2). Posterior distributions were estimated by a General Linear Model regression adjustment [49] based on 0.5% of the simulations: this percentage was chosen as the value at which the marginal densities and modes of the demographic parameters stabilize (S1a and S1b Fig). Model selection was performed by estimating the posterior probability of each model, calculated as the ratio between the marginal density of a particular model and the sum of the marginal densities across all four models. To estimate the power of correctly calling a model, we generated 1,000 pseudo-observed datasets (PODs) under each model and then calculated the fraction of times the correct model had the largest posterior probability. Finally, we followed the approach of Fagundes *et al*. [50] and Veeremah *et al*. [51] to evaluate the corrected posterior probability of each model conditioned on the posterior probabilities of all four models using multivariate kernel estimation. The density estimates were based on the posterior probabilities calculated for 10,000 PODs generated under the model of interest. The bandwidth of the kernel was set equal for all four dimensions (one for each model posterior probability vector) and let take values between 0.01 and 0.2 (S1c Fig).

**Table 1:** Population genetics parameters estimated for the entire gene region and its introns in *C*. *impatiens*.

							Intronic regions							
Gene ^a^	*n* ^b^	*L* ^c^	*ZZ* ^d^	*H* ^e^	*MFDM* ^f^	In. ^g^	*L*_eff_^c^	*P*_I_ ^h^	π ^i^	θ_W_ ^i^	*D*_T_ ^j^	*Div* ^k^	MK ^l^	DoS ^m^
*Cimp*-AT1G07890	116	1125	0.000	0.051	1.0000	5	426	2	0.00008	0.00088	-1.358	0.299	0.4490	-0.563
*Cimp*-AT1G61520	120	510	0.000	0.033	1.0000	2	166	0	0.00000	0.00000	NA	0.199	0.2143	-0.579
*Cimp*-AT1G63440	120	1229	-0.015	0.583	0.8571	3	263	3	0.00263	0.00213	0.408	0.223	0.4539	-0.140
*Cimp*-AT1G69070	110	1748	-0.015	0.688	0.3866	5	451	5	0.00319	0.00207	1.124	0.149	0.5537	-0.125
*Cimp*-AT1G77490	116	1607	-0.015	0.625	1.0000	8	633	6	0.00065	0.00178	-1.390	0.206	0.1729	-0.422
*Cimp*-AT2G15970	120	600	-0.057	0.47	0.7059	2	146	4	0.00954	0.00511	1.667	0.299	0.0398 *	-0.694
*Cimp*-AT2G16500	120	1020	0.028	0.612	0.0336 *	0	NA	NA	NA	NA	NA	NA	1.0000	0.039
*Cimp*-AT2G22590	112	831	0.028	0.61	0.2689	0	NA	NA	NA	NA	NA	NA	0.6825	-0.104
*Cimp*-AT2G31610	120	1000	0.163	0.849	0.1849	3	314	10	0.01073	0.00594	2.009	0.536	0.6918	-0.141
*Cimp*-AT2G36530	114	1224	0.174	0.674	0.1681	6	425	8	0.00477	0.00398	0.487	0.271	0.3029	0.333
*Cimp*-AT2G42540	118	567	0.174	0.62	0.9748	1	278	7	0.00581	0.00471	0.534	0.475	1.0000	0.038
*Cimp*-AT2G44060	118	504	-0.004	0.35	1.0000	0	NA	NA	NA	NA	NA	NA	0.0907	-0.395
*Cimp*-AT4G23850	120	856	0.000	0.171	1.0000	3	268	3	0.00065	0.00209	-1.200	0.171	1.0000	NA
*Cimp*-AT4G29350	116	950	0.000	0.181	1.0000	2	458	12	0.00056	0.00492	-2.305 *	0.225	1.0000	0.013
*Cimp*-AT5G01950	120	1052	0.000	0.017	1.0000	3	244	0	0.00000	0.00000	NA	0.228	0.3654	-0.647
*Cimp*-AT5G11490	120	586	0.000	0.142	0.1176	2	157	4	0.00320	0.00475	-0.629	0.255	0.5055	0.300
*Cimp*-AT5G14420	120	822	-0.066	0.373	0.3361	4	299	3	0.00105	0.00187	-0.764	0.215	1.0000	0.151
*Cimp*-AT5G50100	120	802	0.156	0.718	0.7395	4	289	10	0.01621	0.00645	3.763 **	0.291	1.0000	-0.021
*Cimp*-AT5G51750	114	663	0.008	0.616	1.0000	0	NA	NA	NA	NA	NA	NA	0.0284 *	-0.272
All genes ^n^	117.3 (3.2)	17,696	0.029 (0.076)	0.441 (0.266)	NA	53	4,817	77	0.00394 (0.00480)	0.00311 (0.00212)	0.180 (1.684)	0.269 (0.106)	NA	-0.179 (0.314)

^a^ Name refers to the TAIR-ID of the *A*. *thaliana* orthologue (ATnGnnnnn).

^b^ Number of sequenced haplotypes.

^c^ Number of sequenced sites in the entire gene region (*L*), and number of effective intronic sites (*L*_eff_, where missing data and sites with gaps are excluded).

^d^ Linkage disequilibrium.

^e^ Haplotype diversity.

^f^ Probability of the maximum frequency of derived mutations test; unless specified, values below 0.05 were not significant after correcting for recombination events.

^g^ Number of introns within the sequenced gene region.

^h^ Number of segregating sites.

^i^ Levels of nucleotide diversity, estimated using π and θ_W_.

^j^ Tajima′s *D*.

^k^ Divergence from *A*. *thaliana*.

^l^ Probability of the McDonald-Kreitman test.

^m^ Direction of selection index.

^n^ Mean statistics (SD) and total number of sites across the 19 gene regions.

NA = not available;

* *P* < 0.01;

** *P* < 0.001.

**Table 2:** Population genetics parameters estimated for the entire gene region and its introns in *C*. *resedifolia*.

							Intronic regions							
Gene ^a^	*n* ^b^	*L* ^c^	*ZZ* ^d^	*H* ^e^	*MFDM* ^f^	In. ^g^	*L*_eff_ ^c^	*P*_I_ ^h^	π ^i^	θ_W_ ^i^	*D*_T_ ^j^	*Div* ^k^	MK ^l^	DoS ^m^
*Cres*-AT1G07890	114	1125	0.081	0.797	0.3025	5	455	7	0.00465	0.00290	1.386	0.289	1.0000	-0.083
*Cres*-AT1G61520	120	510	-0.059	0.836	1.0000	2	164	6	0.00701	0.00796	-0.274	0.190	1.0000	-0.015
*Cres*-AT1G63440	116	1229	0.018	0.903	0.4538	3	262	9	0.00861	0.00645	0.818	0.201	1.0000	0.015
*Cres*-AT1G69070	104	1748	0.018	0.907	0.3529	5	492	8	0.00150	0.00305	-1.208	0.138	0.4745	-0.100
*Cres*-AT1G77490	110	1607	0.018	0.793	0.0336	8	738	7	0.00115	0.00180	-0.838	0.266	0.2018	-0.255
*Cres*-AT2G15970	120	600	0.195	0.424	0.2353	2	128	1	0.00063	0.00146	-0.629	0.293	0.1807	-0.286
*Cres*-AT2G16500	112	1020	0.033	0.764	0.4370	0	NA	NA	NA	NA	NA	NA	0.0512	-0.335
*Cres*-AT2G22590	118	831	0.033	0.491	0.0494	0	NA	NA	NA	NA	NA	NA	0.6995	-0.127
*Cres*-AT2G31610	118	1000	0.057	0.333	0.0168	3	319	3	0.00094	0.00176	-0.814	0.510	0.1622	-0.625
*Cres*-AT2G36530	120	1224	0.231	0.668	0.0168	6	424	4	0.00150	0.00176	-0.286	0.265	0.7255	0.102
*Cres*-AT2G42540	120	567	0.231	0.233	0.2689	1	215	2	0.00217	0.00174	0.371	0.461	1.0000	NA
*Cres*-AT2G44060	120	504	0.231	0.049	0.0504	0	NA	NA	NA	NA	NA	NA	0.2500	-0.771
*Cres*-AT4G23850	120	856	-0.162	0.627	0.0672	3	275	2	0.00170	0.00136	0.374	0.159	0.3858	-0.190
*Cres*-AT4G29350	112	950	-0.162	0.913	0.0342	2	502	12	0.00334	0.00489	-0.843	0.247	0.3275	-0.279
*Cres*-AT5G01950	120	1052	0.037	0.79	0.5714	3	246	7	0.00396	0.00531	-0.577	0.232	0.2901	0.354
*Cres*-AT5G11490	120	586	-0.082	0.188	0.0672	2	196	2	0.00034	0.00190	-1.223	0.253	1.0000	-0.200
*Cres*-AT5G14420	120	822	0.167	0.458	1.0000	4	320	1	0.00132	0.00058	1.406	0.216	0.4953	-0.459
*Cres*-AT5G50100	114	802	0.168	0.46	0.0513	4	320	2	0.00032	0.00118	-1.089	0.323	0.1110	-0.692
*Cres*-AT5G51750	114	663	0.039	0.574	1.0000	0	NA	NA	NA	NA	NA	NA	0.0385 *	-0.272
All genes ^n^	116.4 (4.5)	17,696	0.057 (0.122)	0.590 (0.263)	NA	53	5,056	73	0.00261 (0.00248)	0.00294 (0.00219)	-0.228 (0.893)	0.269 (0.101)	NA	-0.234 (0.281)

^a^ Name refers to the TAIR-ID of the *A*. *thaliana* orthologue (ATnGnnnnn).

^b^ Number of sequenced haplotypes.

^c^ Number of sequenced sites in the entire gene region (*L*), and number of effective intronic sites (*L*_eff_, where missing data and sites with gaps are excluded).

^d^ Linkage disequilibrium.

^e^ Haplotype diversity.

^f^ Probability of the maximum frequency of derived mutations test; unless specified, values below 0.05 were not significant after correcting for recombination events.

^g^ Number of introns within the sequenced gene region.

^h^ Number of segregating sites.

^i^ Levels of nucleotide diversity, estimated using π and θ_W_.

^j^ Tajima′s *D*.

^k^ Divergence from *A*. *thaliana*.

^l^ Probability of the McDonald-Kreitman test.

^m^ Direction of selection index.

^n^ Mean statistics (SD) and total number of sites across the 19 gene regions.

NA = not available;

* *P* < 0.01.

### Population genetics analyses

We estimated basic population genetic parameters and performed neutrality tests with the program DnaSP v5.10 [43]. Levels of nucleotide polymorphism θ = 4*N*_e_μ, where μ is the neutral mutation rate and *N*_e_ is the effective population size, were estimated using π (which is the average pairwise difference per base pair; [48]) and θ_W_ (which is solely based on the number of segregating sites; [52]). Divergence *Div* was estimated from the orthologous sequences of *A*. *thaliana*. We also calculated the haplotype diversity, *H* [53], and the linkage disequilibrium *ZZ* statistic [54]. The program TASSEL v3.0 [55] (http://www.maizegenetics.net/tassel, accessed 2015 March 28), was used to evaluate the linkage disequilibrium index *r* ^2^ [56] for each pair of variable sites, and its associated probability calculated by permutations.

Departures from the neutral equilibrium model were tested by the Tajima′s *D*_T_ test [57] and the MFDM test [58], which has the advantage of being free from the confounding signatures of demography.

The program DnaSP was also used to count the number of intraspecific polymorphisms, as well the number of substitutions relative to *A*. *thaliana*, for both synonymous and non-synonymous sites. These numbers were used to assess the selective pressures operating in the genes using the McDonald-Kreitman test [59], and the direction of selection index DoS [60].

### Association analyses

We tested for association between genetic variability and environmental features associated with cold and desiccation stress, i.e. temperature and rainfall precipitation. For each population, we determined mean daily temperature using reconstructed satellite data time series ([61]; Moderate Resolution Imaging Spectroradiometer Land Surface Temperature maps, MODIS LST, available at LPDAAC https://lpdaac.usgs.gov/data_access/data_pool, accessed 2015 March 28; 1000 m resolution data, gap-filled and postprocessed to 250 m resolution) for the period 2003–2010, and mean rainfall precipitation for the period 1981–2010 (aggregated from daily precipitation maps available at ECA&D Europe, http://eca.knmi.nl/, accessed 2015 March 28; 20 km resolution data). Mean values were estimated separately for spring and summer, coinciding with the vegetative and reproductive phase of plants (summer solstice and equinoxes were used as season boundaries; S1 Table). Spring and summer mean temperatures were significantly associated with elevation in both species (Spearman′s ρ < -0.85, *P* < 0.0012, for all correlations), while they were not correlated with geographical coordinates (*P* > 0.58, for all correlations with latitude and longitude), suggesting that they can impose selective pressure on the sampled populations irrespectively from a possible geographic-based population-structure. Similarly, mean rainfall precipitation values were not significantly correlated with longitude, latitude and elevation (*P* > 0.06, for all correlations), nor were they correlated to mean temperature for a given season (*P* > 0.20, for all correlations). Four independent association analyses (one per climatic variable set as predictor) were performed with the mixed linear model approach [62] implemented in TASSEL v3.0 [55], using individual SNP genotypes as dependent variables and the Q-matrix estimated by STRUCTURE to incorporate information about population structure. Since the number of optimal clusters differed depending on the criterion used, we run separate association analyses using Q-matrices estimated for *K* ranging from 2 to 5. Given the small sample size and in order to minimize type II errors, the whole SNP dataset was used for this analysis. To correct for multiple testing, *P* values were calculated based on 100,000 permutations of the dataset and further corrected by the number of independent tests (summer and spring temperature and precipitation, for a total of four tests for each single nucleotide polymorphism, SNP).

## Results

### Gene amplification

Depending on the gene region, we could obtain PCR amplification in 52–60 individuals of both *C*. *impatiens* and *C*. *resedifolia* (mean ± standard deviation was 58.5 ± 0.2). These samples were all successfully sequenced using the high-throughput sequencing approach, and subsequent haplotype reconstruction resulted in a final dataset of 104–120 haplotypes per gene for each species. The sequenced genomic regions had lengths ranging from 504 to 1,748 bp (mean ± SD = 931 ± 345 bp), and contained up to 8 intronic and 9 coding regions. An overview of gene lengths and organization is presented in Tables 1 and 2 and S4 and S5 Tables.

### Population structure and demography

Our analyses revealed weak population structure for both *C*. *impatiens* and *C*. *resedifolia* in the Trentino region.

#### *Cardamine* impatiens

**Fig 2:**
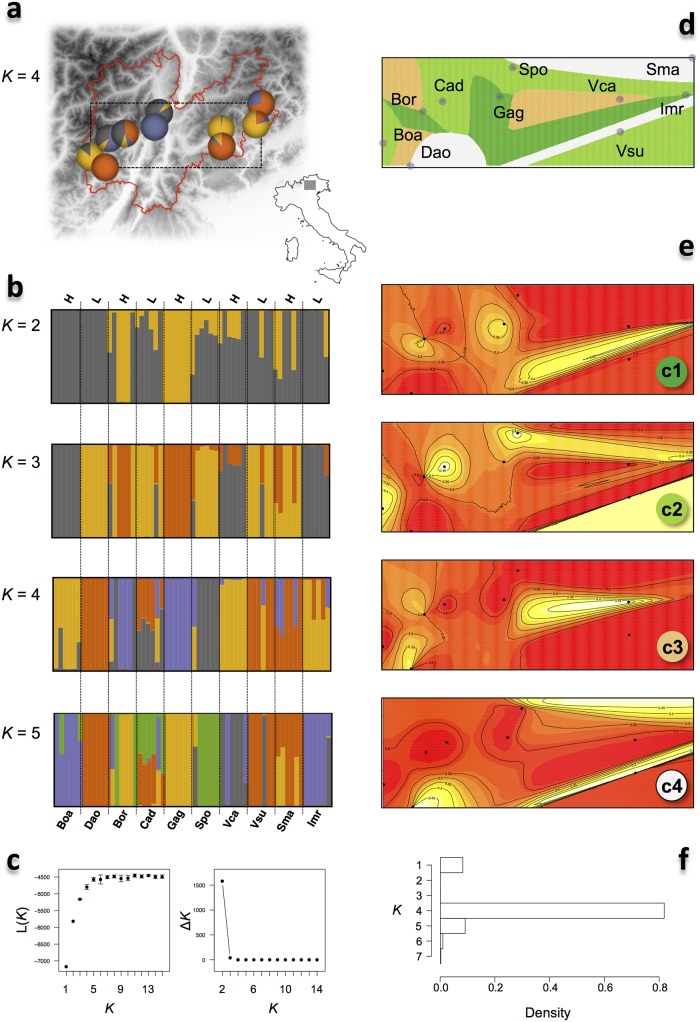
Population structure of *C*. *impatiens* in Trentino. a) Map of Trentino and sampling locations. Pie-charts are colored according to the population ancestry composition for *K* = 4 as inferred by STRUCTURE (for ease of comparison with panel d). b) STRUCTURE analysis: each bar represents an individual, with different colors corresponding to one of the *K* ancestry clusters and length proportional to the assignment to that particular cluster. Individuals are grouped according to the location of sampling (labels are coded as in S1 Table), with H and L indicating high- and low- altitude populations, respectively. Populations are ordered approximately from West to East. c) Average likelihood of the data, L(*K*), and rate of change of the likelihood as a function of the number of clusters *K*. d) Map of the estimated population membership produced by one run of GENELAND for the maximum a-posteriori value of *K* = 4 clusters (see panel f). e) Geographical distribution of the population membership for each of the *K* = 4 clusters (c1, c2, c3, and c4) according to GENELAND. The lighter the shade the higher posterior probability of being assigned to that cluster. f) Number of populations after the burn-in, as simulated by GENELAND.

The approach of Evanno *et al*. [40], based on the results of STRUCTURE [38,39], identified *K* = 2 as the optimal number of clusters (Fig 2a–2c). Such genetic structure did not fit with clear biogeographic patterns. There was however a remarkable asymmetry in the ancestry composition of individuals coming from populations located in the upper margin of the species′ range (H-populations; S1 Table) compared to populations located in the lower margin of the species′ range (L-populations; S1 Table). Specifically, the mean ancestry inferred for the first cluster was of 0.426 in individuals sampled in H-populations compared to a mean of 0.124 in individuals sampled in L-populations. A bootstrap approach that randomly assigned the individual ancestry values (inferred in one of the runs with *K* = 2) to either a H or L population revealed that such difference was not expected by chance (*P* = 0.0005; 10,000 randomizations), and suggests some degree of population differentiation along the altitudinal gradient. When each of the five pairs of adjacent H and L-populations was tested separately, the asymmetry was statistically significant for three comparisons (0.0001 < *P* < 0.05).

The likelihood of the observed genetic variability stabilized at *K* = 5, which then can be considered as the best *K* according to the plateau criterion, with most of the populations mainly belonging to separate clusters. Exceptions were found for few individuals within single populations, and for the Cad population, where all individuals were admixed (Fig 2b). The *K* = 5 ancestry clusters did not identify evident geographical subdivision, describing loose patchy-like population structure across Trentino. The mosaic structure was confirmed by a second analysis, where we made use of spatial information to infer population structure and distribution in a landscape genetics framework [42]. Geneland inferred the presence of *K* = 4 clusters, whose spatial distribution did not correspond to clear geographical features present in the species′ range (Fig 2d–2f).

The overall *F*_ST_ was 0.285, and was higher for pairwise comparisons involving only H-populations (0.336) than only L-populations (0.262), although the difference was not statistically significant (Wilcoxon test, *W* = 72, *P* = 0.1051). We also investigated population differentiation using the actual differentiation index *D*_est_ [45], which has the advantage of not confounding within-group heterozygosity with differentiation. The overall *D*_est_ was 0.064, and significantly higher for pairwise comparisons involving only H-populations (0.089) than only L-populations (0.044; Wilcoxon test, *W* = 84, *P* = 0.0089), consistent with larger differentiation between populations found at the upper distribution range limit. The analysis of the isolation-by-distance model rejected an association between genetic (*F*_ST_) and geographic distance (Mantel test, *P* > 0.056, *r* < 0.226, for both raw and ln-transformed data). The correlation with distance remained not significant when using *D*_est_ (Spearman′s ρ = -0.053, *P* = 0.7303), suggesting that geographic distribution has limited effects on the genetic composition and differentiation of *C*. *impatiens* populations.

**Fig 3:**
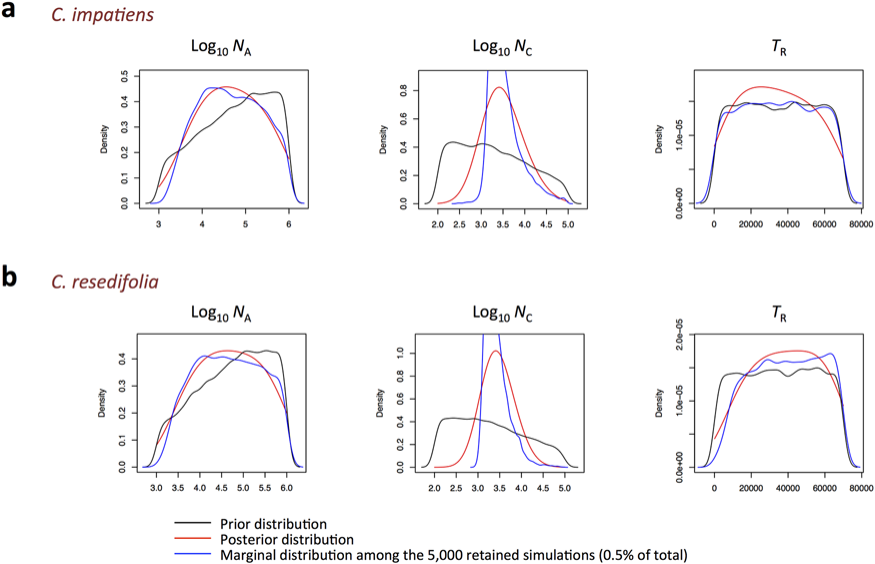
Posterior distributions of the parameters of the demographic model chosen by the ABC approach (population reduction, RED), in *C*. *impatiens* and *C*. *resedifolia*. Ancestral, *N*_A_, and current population size, *N*_C_, are reported in a log_10_ scale; time of the population reduction, *T*_R_, is expressed in number of generations.

We used the ABC approach to infer the most likely demographic history of the Trentino populations among four model scenarios. Power analysis revealed that the constant population size model (CON), the expansion model (EXP), and the reduction model (RED) could be correctly identified as correct models in our ABC analyses (Table 3). The RED model received the highest posterior probability among the four models (Table 3). Posterior distributions of the parameters of this model suggest a current effective population size of 2,613 individuals (95% highest posterior density interval, 95HPD = 387–20,253; Fig 3a and S1d Fig), down from an ancestral size of 37,708 (95HPD = 2,466–706,692), and time of reduction set at 25,391 generations ago (95HPD = 1,505–62,272).

**Table 3:** Results of model fitting and model choice of the demographic models tested in *C*. *impatiens* and *C*. *resedifolia*.

Model ^a^	MD ^b^	*P* ^c^	p*P* ^d^	Power ^e^	p*P*_C_ ^f^
*C*. *impatiens*					
CON	75.4	0.846	0.0178	0.77	0.0715
BOT	4.9	0.665	0.0012	0.20	0.1886
EXP	59.7	0.155	0.0141	0.56	0.0958
RED	4088.9	0.846	0.9669	0.31	0.6442
*C*. *resedifolia*					
CON	3917.1	0.999	0.0242	0.77	0.0192
BOT	337.3	1.000	0.0021	0.25	0.0118
EXP	58504.8	1.000	0.3611	0.55	0.0157
RED	99260.9	0.996	0.6126	0.32	0.9534

^a^ Demographic model: CON = constant populations size; BOT = population size bottleneck; EXP = population size expansion; RED = population size reduction.

^b^ Marginal density.

^c^ Fraction of the retained simulations (*n* = 5,000, corresponding to 0.5% of the total simulations) with a likelihood smaller or equal to that obtained for the observed data (Model fit).

^d^ Posterior probability, estimated as ratio between the marginal density (MD) of a model and sum of the marginal densities across models.

^e^ Fraction of pseudo-observed datasets generated under the model (PODs) for which such model was correctly identified as the best based on posterior probability (p*P*). Random expectation is 1/(no. of models) = 0.25.

^f^ Posterior probability corrected using multivariate kernel density estimation with bandwidth = 0.1.

#### *Cardamine* resedifolia

**Fig 4:**
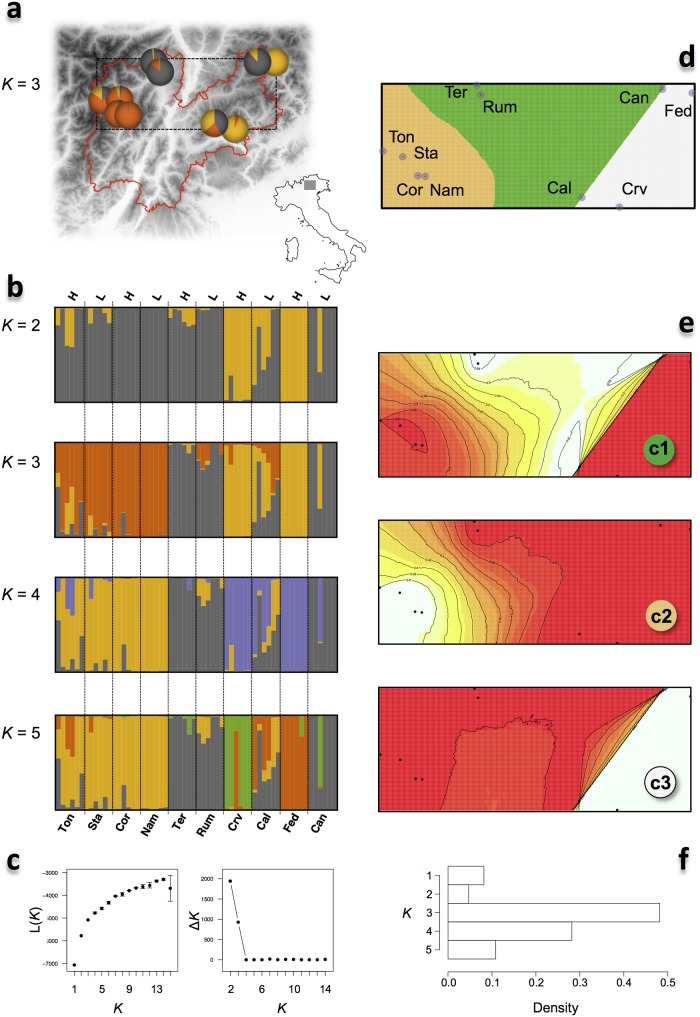
Population structure of *C*. *resedifolia* in Trentino. a) Map of Trentino and sampling locations. Pie-charts are colored according to the population ancestry composition for *K* = 3 as inferred by STRUCTURE (for ease of comparison with panel d). b) STRUCTURE analysis: each bar represents an individual, with different colors corresponding to one of the *K* ancestry clusters and length proportional to the assignment to that particular cluster. Individuals are grouped according to the location of sampling (labels are coded as in S1 Table), with H and L indicating high- and low- altitude populations, respectively. Populations are ordered approximately from West to East. c) Average likelihood of the data, L(*K*), and rate of change of the likelihood as a function of the number of clusters *K*. d) Map of the estimated population membership produced by one run of GENELAND for the maximum a-posteriori value of *K* = 3 clusters (see panel f). e) Geographical distribution of the population membership for each of the *K* = 3 clusters (c1, c2, and c3) according to GENELAND. The lighter the shade the higher posterior probability of being assigned to that cluster. f) Number of populations after the burn-in, as simulated by GENELAND.

In this species the approach of Evanno et al. [40] identified K = 2 clusters that contributed differently to the ancestry composition of high and low populations (Fig 4a–4c; S1 Table). The mean ancestry for the first cluster was of 0.448 in H-populations and of 0.128 in L-populations (*P* = 0.0003; 10,000 randomizations). This asymmetry was confirmed for three out of the five pairs of adjacent H and L-populations (0.0001 < *P* < 0.04). In *C*. *resedifolia* the likelihood of the observed genetic variability did not reach a plateau at the increase of *K*. Nonetheless, an interesting pattern emerged with *K* = 3 clusters, which defined a group of four western populations having very similar ancestry, two “central” populations with genetic ancestry similar to the easternmost population, and three eastern populations in which the third cluster contributed heavily. The overall structure pattern was confirmed at *K* = 4, when a fourth cluster was detected with extremely low frequency (0.001 in a single population), and at *K* = 5, when an additional cluster separated one of the eastern populations. The spatial structure was strongly supported by the results of the landscape genetics approach of Geneland, which inferred *K* = 3 clusters describing a longitudinal population subdivision across Trentino (Fig 4d–4e).

The overall differentiation as measured by *F*_ST_ was of 0.289 and was significantly higher for pairwise comparisons involving only H-populations (0.370) than only L-populations (0.215; Wilcoxon test, *W* = 85, *P* = 0.007). This difference was confirmed by *D*_est_ (overall 0.023; 0.036 for H-populations, and 0.013 for L-populations, Wilcoxon test, *W* = 90, *P* = 0.0028), and was consistent with less gene flow among populations found at high altitudes than among populations found at low altitude. Interestingly and opposite to what observed for *C*. *impatiens*, in *C*. *resedifolia* there is isolation-by-distance (Mantel test, *P* < 0.011, *r* > 0.491, for both raw and ln-transformed data).

The ABC approach inferred in the RED model the most likely demographic history of the Trentino populations (Table 3 and S1c Fig). Posterior distributions of the parameters suggest a current effective population size of 2,524 individuals (95% highest posterior density interval, 95HPD = 529–12,458; Fig 3b and S1d Fig), down from an ancestral size of 42,477 (95HPD = 2,466–784,260) about 45,061 generations ago (95HPD = 8,179–67,893).

### Nucleotide polymorphism in *C*. *impatiens* and *C*. *resedifolia*

Analysis of basic population genetics statistics revealed little differences between patterns of molecular evolution of *C*. *resedifolia* and *C*. *impatiens* (Tables 1 and 2 and S4 and S5 Tables). Haplotype diversity was not statistically different between the two species (Wilcoxon test, W = 122, *P* = 0.09), and levels of nucleotide polymorphism, measured as π and θ_W_, were also similar in *C*. *resedifolia* and *C*. *impatiens* in both the coding and non-coding regions (Wilcoxon test, *P* > 0.18, for all comparisons). Taken together, and assuming equal mutation rates, these results suggest that the two species have comparable effective population size *N*_e_. This conclusion, however, is in part contradicted by two further observations. The first is based on the analysis of haplotype diversity values estimated separately in each of the sampled populations: in this case the across-populations mean was significantly larger in *C*. *resedifolia* than in *C*. *impatiens* (0.407 *vs*. 0.254, Wilcoxon test, W = 265, *P* = 0.0136). The contrast to the overall haplotype diversity analysis is consistent with relatively higher within-population and lower-between genetic homogeneity in *C*. *impatiens*, as supported by a significantly larger differentiation index *D*_est_ in *C*. *impatiens* than in *C*. *resedifolia* (average ± SD was 0.064 ± 0.039 in *C*. *impatiens* and 0.023 ± 0.015 in *C*. *resedifolia*, Wilcoxon test, W = 1816, *P* < 10^-10^). The second observation is based on the analysis of genetic association between polymorphic sites. The two species had levels of intragenic linkage disequilibrium (*ZZ*) that were not statistically significant (*P* = 0.084). However, the degree of linkage disequilibrium *r* ^2^ over all pairwise comparisons of the concatenated dataset was significantly lower in *C*. *resedifolia* than in *C*. *impatiens* (0.031 *vs*. 0.048, Wilcoxon test, W = 404615952, *P* < 10^–10^), suggesting long-range linkage disequilibrium in the latter, possibly associated with higher levels of selfing.

The analysis of the polymorphism frequency spectrum (which was limited to the non-coding portions of the genes) revealed fairly large heterogeneity across genes in both species, with both positive and negative Tajima′s *D*_T_ values producing a mean of *D*_T_ across gene close to zero (Tables 1 and 2). In *C*. *impatiens*, the Tajima test [57] revealed two genes with significant departures from the neutral model. Visual inspection of the alignments revealed that in general very negative and positive *D*_T_ values were associated with, respectively, rare or common divergent haplotypes. In fact, negative *D*_T_ values were not associated with low polymorphism (θ) levels, which rules against recent, rare, variants typical of recent positive selection (selective sweep, [63]). Rather, this observation confirmed the presence of diverged haplotypes restricted to single populations, which thus have low haplotype diversity but contribute to produce overall high haplotype diversity across populations in *C*. *impatiens*. Such variance in the frequency spectra is also present in *C*. *resedifolia*, although significantly less than in *C*. *impatiens* (*F* test for unequal variances, *P* = 0.0267), and according to Tajima′s test no gene departed from neutrality in this species. To further test for departures from the neutral model of evolution, we employed the maximum frequency of derived mutations (MFDM) test proposed by Li [58]. Only a single gene had a nominal *P* value lower than 0.05 in *C*. *impatiens*, which however became not significant after multiple testing correction. In *C*. *resedifolia*, no gene departed from neutral expectations according to the MFDM test. Thus, neutrality tests did not provide evidence for the action of positive selection in any of the genes analyzed, suggesting that natural selection may have not occurred in these genes during the recent history of the two species. Consistently, there were no difference in levels of nucleotide polymorphism, haplotype diversity, linkage disequilibrium (*ZZ*), and Tajima′s *D* between candidate genes and neutral genes in neither *C*. *impatiens* and *C*. *resedifolia* (Wilcoxon tests, *P* > 0.436, for all comparisons), indicating similar rates of evolution in the two classes of genes.

We next investigated whether the joint analysis of polymorphism and divergence data of coding regions provided patterns compatible with the action of positive selection (Tables 1 and 2). The McDonald-Kreitman test [59] did not detect any gene showing evidence of positive selection, thus confirming the polymorphism-based analyses. Consistently, the average DoS statistic [60] was negative, indicating prevalence of purifying selection across genes.

### Association analyses

Populations sampled at their upper and lower altitudinal bounds experience contrasting temperature regimes (S1 Table). However, the association analysis revealed no single nucleotide polymorphism (SNP) associated with either mean spring or summer temperatures (Table 4). In contrast, there were several SNPs associated with the mean spring and summer precipitation (Table 4; for *C*. *impatiens*: range of mean precipitation was 213–276 mm in spring and 224–346 in summer; for *C*. *resedifolia*: ranges were 200–296 in spring and 235–346 in summer; S1 Table). In *C*. *impatiens*, a total of 23 SNPs (hereafter referred to as SNPs^rain^) distributed in 9 genes were significantly associated with variation in mean spring rainfall precipitation. The number of SNPs^rain^ changed depending on the number of genetic clusters *K* used to define the Q-matrix, but most were confirmed for different *K* values and all but one were found associated at *K* = 3. Twelve of these SNPs^rain^ were also significantly associated to variation in mean summer rainfall precipitation. There was no significant enrichment in candidate genes among those harbouring SNPs^rain^ (Fisher′s exact test, *P* = 1).

**Table 4:** Polymorphisms associated to climatic variables.

			Frequency in *C*. *impatiens* populations ^d^										Rainfall ^e^	
Gene ^a^	Pos ^b^	SNP type ^c^	Boa	Dao	Bor	Cad	Gag	Spo	Vca	Vsu	Imr	Sma	Spring	Summer
AT1G63440 *nc*	1086	*syn*	0.25	0	0.33	0	1.00	1.00	0.17	0.17	0	0	2, 3, 5	-
AT1G77490 *c*	1378	*nonsyn* (A/T)	0	0	0.33	0.75	1.00	0.42	0	0	0	0	2, 3, 4, 5	2, 3
AT2G16500 *c*	829	*syn*	0.08	0	1.00	0.50	1.00	1.00	0.50	0.17	0.17	0	3, 5	-
AT2G16500 *c*	982	*nonsyn* (F/L)	0.08	0	1.00	0.50	1.00	1.00	0.50	0.17	0.17	0	3, 5	-
AT2G22590 *nc*	326	*nonsyn* (K/P)	0	0	0.83	0.25	1.00	0.75	0	0	0	0	2, 3, 5	2, 3, 5
AT2G22590 *nc*	327	*nonsyn* (K/P)	0	0	0.83	0.25	1.00	0.75	0	0	0	0	2, 3, 5	2, 3, 5
AT2G22590 *nc*	328	*nonsyn* (K/P)	0	0	0.83	0.25	1.00	0.75	0	0	0	0	2, 3, 5	2, 3, 5
AT2G22590 *nc*	567	*nonsyn* (D/G)	0	0	0.67	0.25	1.00	0.75	0	0	0	0	2, 3, 5	2, 3, 5
AT2G31610 c*	127	*noncod*	0.83	0	0.50	1.00	0	0.83	0	0	0	0	2, 3, 5	-
AT2G36530 *c*	338	*noncod*	1.00	0	1.00	1.00	1.00	0.83	0	0	0	0	2, 3, 5	2, 3, 5
AT2G36530 *c*	823	*syn*	1.00	0	1.00	1.00	1.00	0.83	0	0	0	0	2, 3, 5	2, 3, 5
AT2G36530 *c*	971	*noncod*	1.00	0	0.33	1.00	1.00	0.83	0	0	0	0	2, 3, 4, 5	2, 3, 5
AT2G36530 *c*	972	*noncod*	1.00	0	0.33	1.00	1.00	0.83	0	0	0	0	2, 3, 4, 5	5
AT2G42540 *c*	128	*noncod*	0	1.00	0.67	0.08	1.0	0.08	0.08	0.08	1.00	1.00	3	-
AT2G42540 *c*	234	*noncod*	1.00	0	0.33	0.67	0	0.83	0.83	0.83	0	0	3	-
AT2G42540 *c*	273	*noncod*	1.00	0	0.33	0.67	0	0.83	0.83	0.83	0	0	3	-
AT2G42540 *c*	277	*noncod*	1.00	0	0.33	0.67	0	0.83	0.83	0.83	0	0	3	-
AT2G42540 *c*	332	*noncod*	0	0	0.67	0.08	1.00	0.08	0.08	0.08	1.00	1.00	2	4, 5
AT2G42540 *c*	364	*noncod*	1.00	0	0.33	0.67	0	0.83	0.83	0.83	0	0	2, 3, 5	-
AT2G42540 *c*	517	*nonsyn* (E/D)	1.00	0	0.33	0.67	0	0.83	0.83	0.83	0	0	2, 3, 5	-
AT5G50100 *nc*	34	*nonsyn* (N/K)	0	1.00	0	0	0	0	0	0.83	0	1.00	3, 5	3, 5
AT5G50100 *nc*	303	*syn*	0	0	0.67	1.00	1.00	0.83	0	0	0	0	2, 3, 5	2, 3, 5
AT5G51750 *nc*	186	*nonsyn* (A/V)	0.50	0	0.33	0	1.00	0.50	0	0	0	0	2, 3, 5	-
			**Frequency in *C*. *resedifolia* populations** ^**d**^											
			**Nam**	**Cor**	**Ton**	**Sta**	**Rum**	**Ter**	**Cal**	**Crv**	**Can**	**Fed**		
AT1G07890 c*	747	*nonsyn* (E/D)	0	0	0	0	0.58	0.75	0	0	0	0	2, 5	-
AT1G61520 *c*	291	*noncod*	0	0	0.42	0	0	0	0	0	0.83	0	3, 4	2, 3, 4, 5
AT2G15970 *c*	200	*noncod*	0	0	0	0	0.83	0.33	0	0	0	0	2, 5	-
AT2G16500 *c*	57	*nonsyn* (P/L)	0	0	0	0	0	0	0	0	0.80	0	3, 4	2, 3, 4, 5
AT2G29350 c*	242	*noncod*	0.17	0.40	0.75	0.33	0	0	0.20	0	0	0.17	2, 3, 4, 5	3, 4
AT5G01950 *nc*	685	*noncod*	0	0	0	0	0	0	0	0	0.83	0	3, 4	2, 3, 4, 5

^a^ TAIR-ID of the *A*. *thaliana* orthologue; *c* = candidate gene (* after Ometto *et al*. 2012); *nc* = non-candidate (neutral) gene.

^b^ Relative position of the SNP in the alignment.

^c^ SNPs could be at non-coding intronic sites (*noncod*), synonymous sites (*syn*), or non-synonymous sites (*nonsyn*). For the latter, the two alternative aminoacids (abbreviated using the one letter standard code) are given in brackets.

^d^ Frequency of the SNP minor allele in each population. Populations are sorted along the longitudinal gradient and identified using the codes given in S1 Table.

^e^ Results of the association test (performed by TASSEL) between mean rainfall precipitation during spring and summer and frequency of the allelic variants at the SNP: numbers identify the population clusters *K* estimated by STRUCTURE at which the association was statistically significant (*P* < 0.05 after correcting for multiple testing; *K* tested ranged from 2 to 5).

The results were very similar in *C*. *resedifolia*, where six SNPs^rain^ were associated with mean spring (*n* = 6) and summer (*n* = 4) rainfall precipitation and distributed over six genes (Table 4). All such SNPs^rain^ were confirmed for different *K* values and were located in six genes. Also in this species there was no significant enrichment in candidate genes among those harbouring SNPs^rain^ (Fisher′s exact tets, *P* = 0.177).

## Discussion

### Population structure, demographic history, and local adaptation in *C*. *impatiens*

In *C*. *impatiens*, two observations point to a lack of appreciable population structure across the sampling transect. The first is the absence of a consensus in the number of genetic clusters identified by the different approaches used in this study. For instance, STRUCTURE inferred the presence of two genetic clusters, while Geneland detected four clusters (Fig 2). The increase in the number of clusters underpinned additional population substructuring, with each population being generally represented by one or two clusters. Notably, whenever two genetic clusters were present in a population, they were usually found in equal amounts in all or most of its individuals, consistent with (recent) admixture of two lineages with distinct ancestry. Overall, this pattern indicates high within- and low between-population genetic similarity, and suggests local founder events possibly associated with the high selfing rate and/or inbreeding reported for this species [64]. The results of our analyses are overall consistent with this hypothesis. First, haplotype diversity was much higher when pooling all individuals across populations than for each population independently. Second, the results of the population structure analyses revealed a weak signature of population differentiation associated with the elevation gradient, with *F*_ST_ values suggesting that on average populations found at higher altitudes exchange less migrants than those at lower altitudes. This scenario makes sense if we assume that gene flow may be facilitated along valleys, while H-populations constitute derived isolated populations that have less chance to enter in contact one another due to geographical constraints. On the other hand three observations, namely the patchy population structure of *C*. *impatiens*, the fact that the same genetic clusters could be found in distant populations, and the absence of isolation-by-distance, together suggest that this species may have experienced frequent episodes of long-distance dispersal. This observation is in line with the documented, although relatively low, endozoochorous dispersion of *C*. *impatiens* by red deer [65]. This scenario is also compatible with the results of the ABC simulations, which indicated a model with population size reduction as the most plausible demographic scenario for the Trentino populations. This reduction may coincide with an invasion of the Alpine region by this species, such that different lineages have independently occupied low altitude valleys and subsequently entered into contact. Thus, the haplotype structure is more likely due to recent admixture events, rather than weak incomplete bottlenecks [53].

Signatures of ecological differentiation, in the form of positive selection and local adaptation, were absent at the single-gene level. Likewise, association analyses between single SNPs and environmental gradients provided only marginal evidence for local adaptation. There were 23 SNPs^rain^ significantly associated with mean spring and/or summer rainfall precipitation, nine of which coded for amino acid changes. Interestingly, four of the non-synonymous SNPs^rain^ were found in genes (AT2G16500, AT2G31610, AT2G36530, AT2G42540) involved in salt stress response (S2 Table), a process which is functionally associated with water availability/deprivation, and/or to cold stress. While appealing, however, the possibility that all the alleles at the SNPs are involved in local adaptation deserves some caution. First, the rainfall data had rather low resolution (~20 km), such that the proximity of the populations occasionally prevented the assignment of population-specific mean rainfall values (for example, Gag and Spo are assigned very similar values). Second, it also cannot be excluded that these genes are located in the same chromosome and be in linkage disequilibrium one another, since in *A*. *thaliana* they all are found in chromosome 2.

### Population structure, demographic history, and local adaptation in *C*. *resedifolia*

Compared to *C*. *impatiens*, population structure was more pronounced in *C*. *resedifolia* despite the good colonization ability and partial anemophylous dispersal syndrome reported for diaspores of this species [66]. The two genetic clusters identified as the most likely level of subdivision by STRUCTURE had opposing frequencies in the H- and L-populations. This asymmetry underpinned a larger genetic differentiation between H-populations than between L-populations, suggesting that population at high altitudes are more isolated compared to those found at lower altitudes. Gene flow among L-populations may be facilitated due to their closer proximity in terms of actual path distance. In contrast, H-populations are not directly in contact and gene flow occurs only as “mediated” by nearby L-populations. Thus, one may envision a scenario where high altitude populations originated from a large population at the lower bound of the species distributional range. This makes sense considering that the present distribution of the species follows the occupation of new habitats after the last glaciation maximum (LGM), with warmer temperatures allowing the colonization of higher altitude habitats. Interestingly, mean polymorphism estimated in L-populations (θ_W_ = 0.0017; π = 0.0019) was larger than in H-populations (θ_W_ = 0.0017; π = 0.0016; Wilcoxon tests, *P* = 0.032 for θ_W_, *P* = 0.222 for π), consistent with a larger effective population size in the former.

The results of STRUCTURE and Geneland revealed a population subdivision defining a clear longitudinal pattern of population structure. Notably, this genetic differentiation is accompanied by a significant correlation between population-specific polymorphism, θ_W_, and longitude in H-populations (Spearman′s correlation, ρ = -1, *P* = 0.0167, *R* ^2^ = 0.91; partial correlation when correcting for elevation, ρ = -0.9, *P* = 0.083; for L-populations and all populations combined, *P* > 0.31; similar results were obtained if π or haplotype diversity were used instead). This suggests that western H-populations harbor more genetic diversity than eastern populations, possibly because of a longer establishment allowing for higher gene flow with the neighboring populations, thus increasing the heterozygosity of the populations. This hypothesis is in part supported by a phylogeographic analysis of *C*. *resedifolia* across its distributional range [67], which includes mountain regions going from the Pyrenees to the Alps and the Balkans [29,67], and is thus only marginally covered by the populations sampled for this study. Lihova *et al*. [67] reported weak population subdivision across the species distributional range, with two main groupings distributed over a large portion of the species range, a pattern interpreted as the result of fair gene flow among regions allowing the persistence of a widespread gene pool. Interestingly, one of the two main genetic groups was found to be further split into four subgroups corresponding to distinct and contiguous geographic areas of the Alps, namely the south- western Alps, northern Alps, and the Hohe Tauern (Austria, Central-eastern Northern Alps). A closer inspection of their clustering results (Fig. five of [67]) revealed a fine substructure at *K* = 10 where genetic clusters identify sequential populations along the Western, and especially Eastern Alps (which include Trentino). The population structure thus conforms to that of a species experiencing fair degree of gene flow between populations, which are however sufficiently isolated (given their habitat preference) to lead to significant genetic isolation-by-distance. The results of the demographic analysis suggested a quite severe population reduction around 45,000 years ago (assuming a generation per year for this species, consistently to the observed time to first flower; Varotto, unpublished results), thus before the LGM (around 16,000 years ago). Therefore, the Trentino population seems to have only marginally suffered the habitat contraction following the LGM, either because this population was sampled very close to the putative southern refugia [68] and thus retained most of the ancestral genetic diversity, or/and because central Alpine *C*. *resedifolia* populations may indeed contain diversity from multiple ancestral peripheral populations [67] to which the western contributed more (see above).

In *C*. *resedifolia* no gene departed from neutrality, suggesting that the candidate genes did not undergo episodes of positive selection in the recent history of the species, neither at the species nor at the population level. The association analyses revealed six SNPs^rain^ in as many genes associated with mean rainfall precipitation, one of which was located at a non-synonymous site of the salt stress responsive gene AT1G07890. However, as mentioned above, rainfall data suffer from low resolution, thus preventing robust conclusions about the significance of this result. For instance, the two SNPs^rain^ associated with mean spring rainfall had their minor allele at high frequency in two populations, Rum and Ter, that had the same rainfall values and were assigned to the same cluster in the population structure analysis (Fig 4). This suggests that, despite using clustering information, TASSEL was not able to completely remove the effects of population structure from the analysis, possibly biasing the association between genetic and climatic variability.

## Conclusions

Several reasons may explain the absence of signatures of local adaptation in *C*. *resedifolia* and *C*. *impatiens* among the genes considered in this study. The first is that, despite H- and L-populations experienced contrasting environmental (selective) regimes, we did not have enough power to detect departures from neutrality due to a partial coverage of the genetic diversity of the species. Our samples cover only a limited portion of the species distribution range, and a broader sampling (including more extreme environments) may provide precious genetic variants involved in local adaptation. In addition, the presence of gene flow among populations may also affect the fixation probability of the beneficial allele by introducing less fit migrant alleles (e.g. [69]). The second is that the climatic variables considered do not affect the species′ fitness strongly enough to drive adaptation at a local scale. For instance, phenotypic plasticity may allow the plants to readily grow across a wide range of environmental conditions (e.g. [70]), actually eliminating the need of alternative genetic variants. A third reason is the number of analyzed genes, which represent only a limited set of those present in *Cardamine*. Adding more genes (both neutral and candidate) would definitely increase the chance of finding the genetic basis for local adaptation in these species. Moreover, the landscape genomic analyses may have lacked enough power due to the low number of individuals sampled in each population, although this may not have been a limited factor when contrasting low- versus high-altitude populations. A final possibility rests upon the strong link between efficiency of selection and the demographic processes and properties typical of the two species. Depending on the strength of local selective pressures, local adaptation may in fact be hindered by the reduction in the efficiency of selection due to small effective population size [71]. This reduction may result from structure [72] or from demographic events like the population size reduction suggested by our demographic analyses. The lack of signatures of positive selection in any of the genes, including the candidate ones, partly contrasts with the results of our previous analysis [31], which was based on the analysis of the codon substitution models on the phylogenetic tree [73]. The reason of this discrepancy may be due to the different time scales upon which the two approaches are used, whereby the signatures of ancient positive selection along the *C*. *resedifolia* lineage may have been captured only by the codon substitution model approach. Additional population genomics approaches will in the future certainly help identifying possible targets of positive selection and local adaptation in the two species.

## Supporting Information

S1 Fig Results of the four demographic models investigated by Approximate Bayesian Computation (ABC) for *Cardamine impatiens* and *C*. *resedifolia*. For each demographic model we report the marginal densities (a) and modes (b), the bandwidth of the kernel (c), and the posterior probabilities of each of the model parameters (d).
(PDF)

S1 Table Sampling localities. (PDF)

S2 Table Gene function as inferred from the *A*. *thaliana* orthologue. (PDF)

S3 Table Primers used to amplify the genes used in the study. (PDF)

S4 Table Polymorphism in the coding regions of *C*. *impatiens*. (PDF)

S5 Table Polymorphism in the coding regions of *C*. *resedifolia*. (PDF)
