# Supplementary material for: Demographic History, Population Structure, and Local Adaptation in Alpine Populations of Cardamine impatiens and Cardamine resedifolia
Source: PLoS One. 2015 May 1;10(5):e0125199. doi: 10.1371/journal.pone.0125199 (PMC4416911; doi:10.1371/journal.pone.0125199)

Figure S1. Results of the four demographic models investigated by Approximate Bayesian Computation (ABC) for *Cardamine impatiens* and *C. resedifolia*. For each demographic model we report the marginal densities (a) and modes (b), the bandwidth of the kernel (c), and the posterior probabilities of each of the model parameters (d).

Cardamine impatiens

a

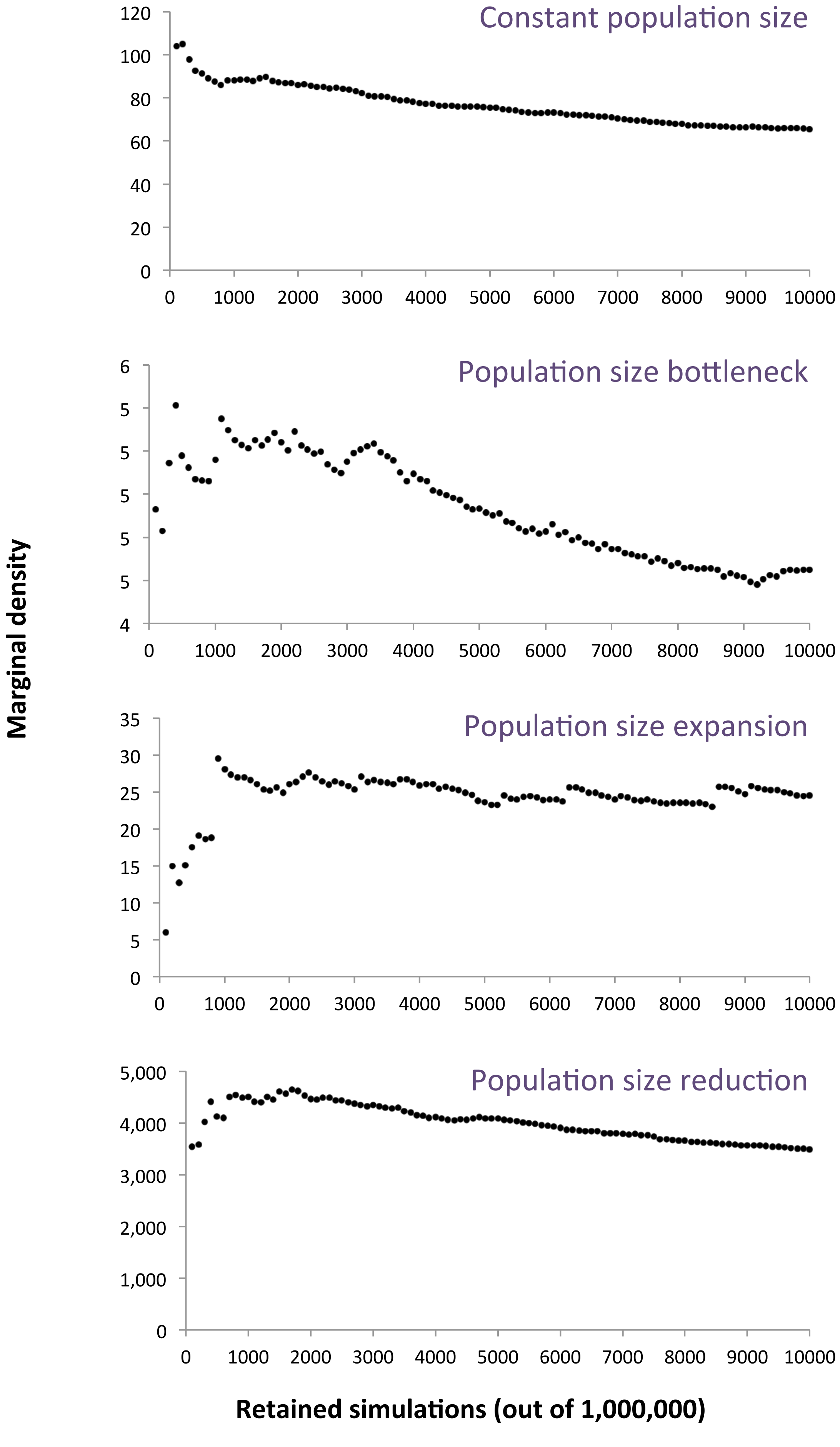

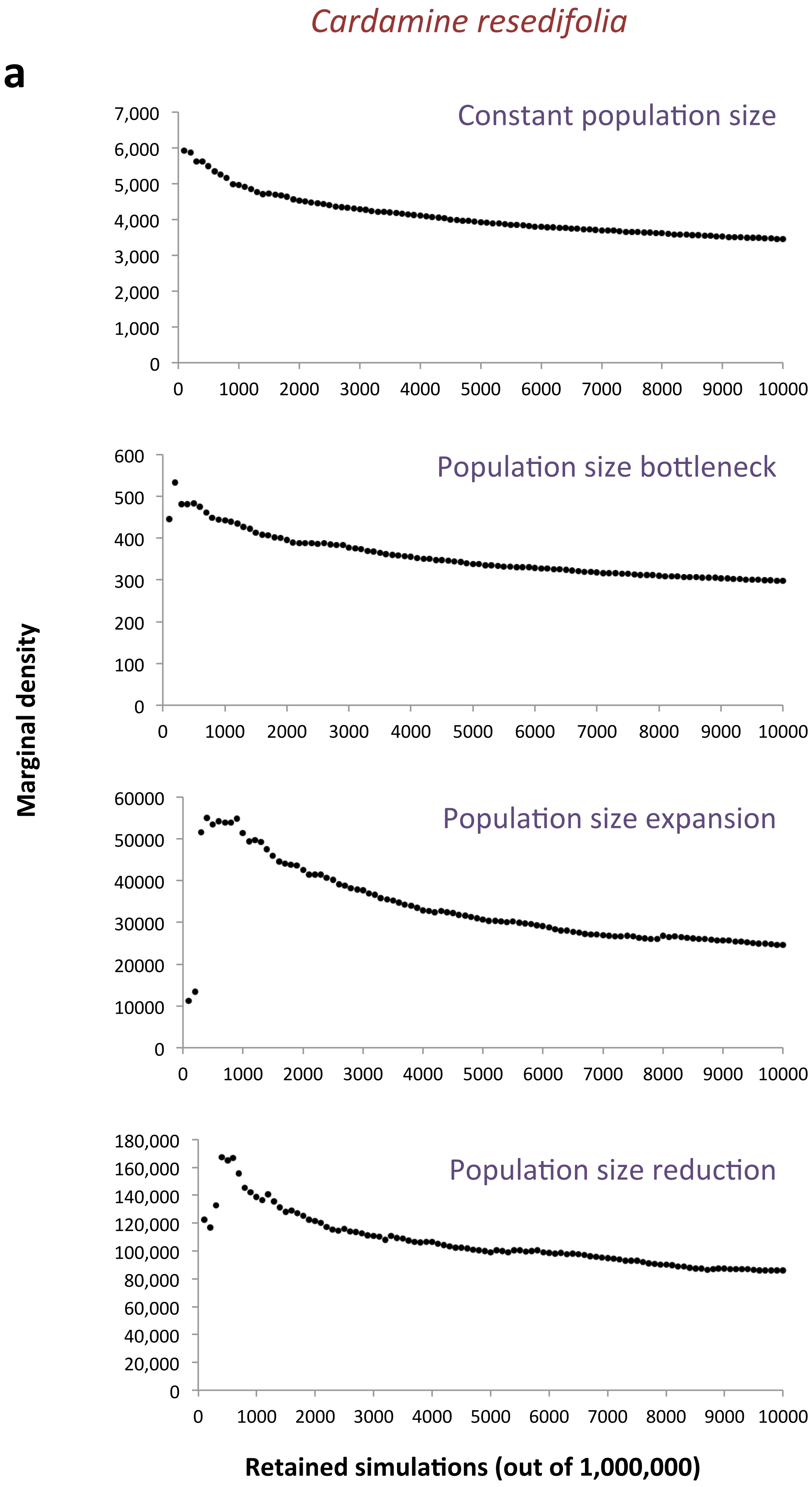

Cardamine resedifolia
a
Constant population size
7,000
6,000
5,000
4,000
3,000
2,000
1,000
0
0 1000 2000 3000 4000 5000 6000 7000 8000 9000 10000
Population size bottleneck
600
500
400
300
200
100
0
0 1000 2000 3000 4000 5000 6000 7000 8000 9000 10000
Population size expansion
60000
50000
40000
30000
20000
10000
0
0 1000 2000 3000 4000 5000 6000 7000 8000 9000 10000
Population size reduction
180,000
160,000
140,000
120,000
100,000
80,000
60,000
40,000
20,000
0
0 1000 2000 3000 4000 5000 6000 7000 8000 9000 10000
Marginal density
Retained simulations (out of 1,000,000)

b *Cardamine impatiens*

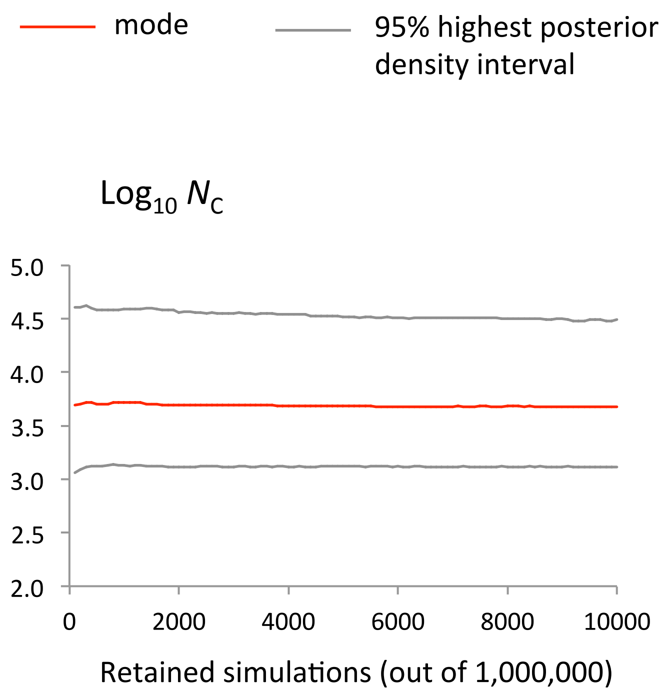

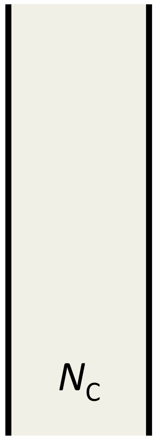

Constant population size (CON)

N_C = Current population size

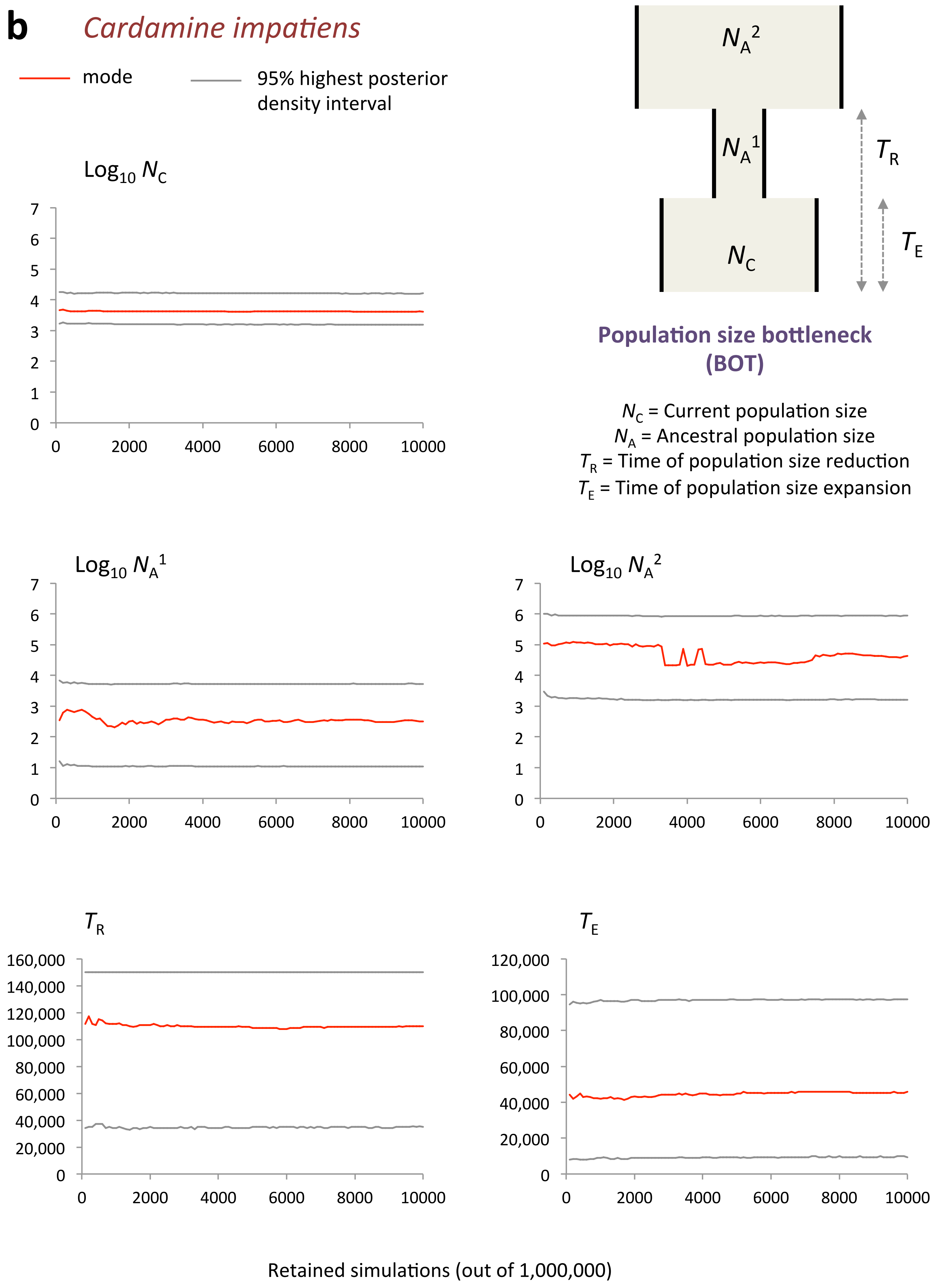

b
Cardamine impatiens
mode
95% highest posterior density interval
Log10 NC
NA2
NA1
NC
TR
TE
Population size bottleneck (BOT)
NC = Current population size
NA = Ancestral population size
TR = Time of population size reduction
TE = Time of population size expansion
Log10 NA1
Log10 NA2
TR
TE
Retained simulations (out of 1,000,000)

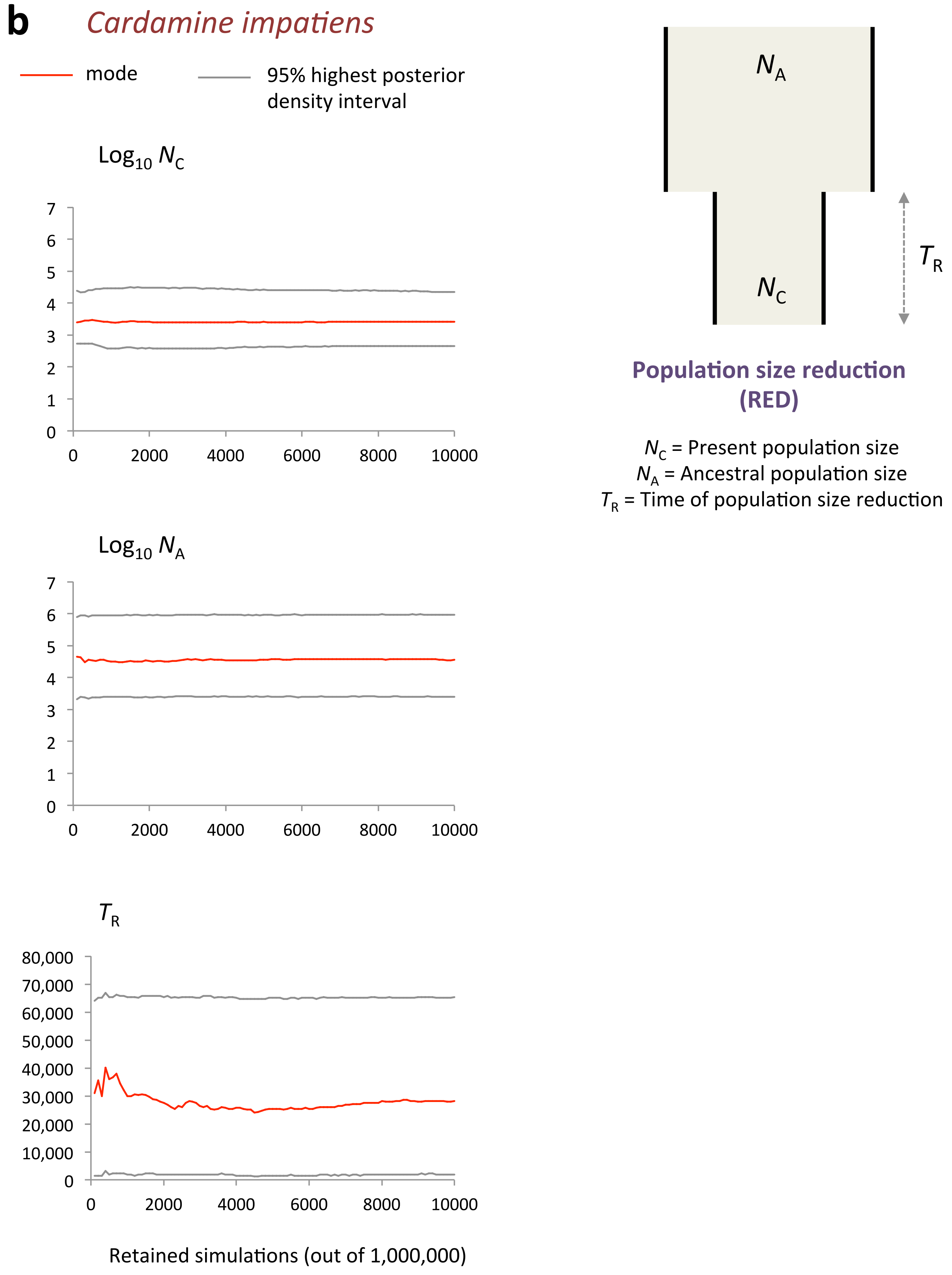
b
Cardamine impatiens
mode
95% highest posterior density interval
Log10 NC
7
6
5
4
3
2
1
0
0
2000
4000
6000
8000
10000
Log10 NA
7
6
5
4
3
2
1
0
0
2000
4000
6000
8000
10000
TR
80,000
70,000
60,000
50,000
40,000
30,000
20,000
10,000
0
0
2000
4000
6000
8000
10000
Retained simulations (out of 1,000,000)
NA
NC
TR
Population size reduction (RED)
NC = Present population size
NA = Ancestral population size
TR = Time of population size reduction

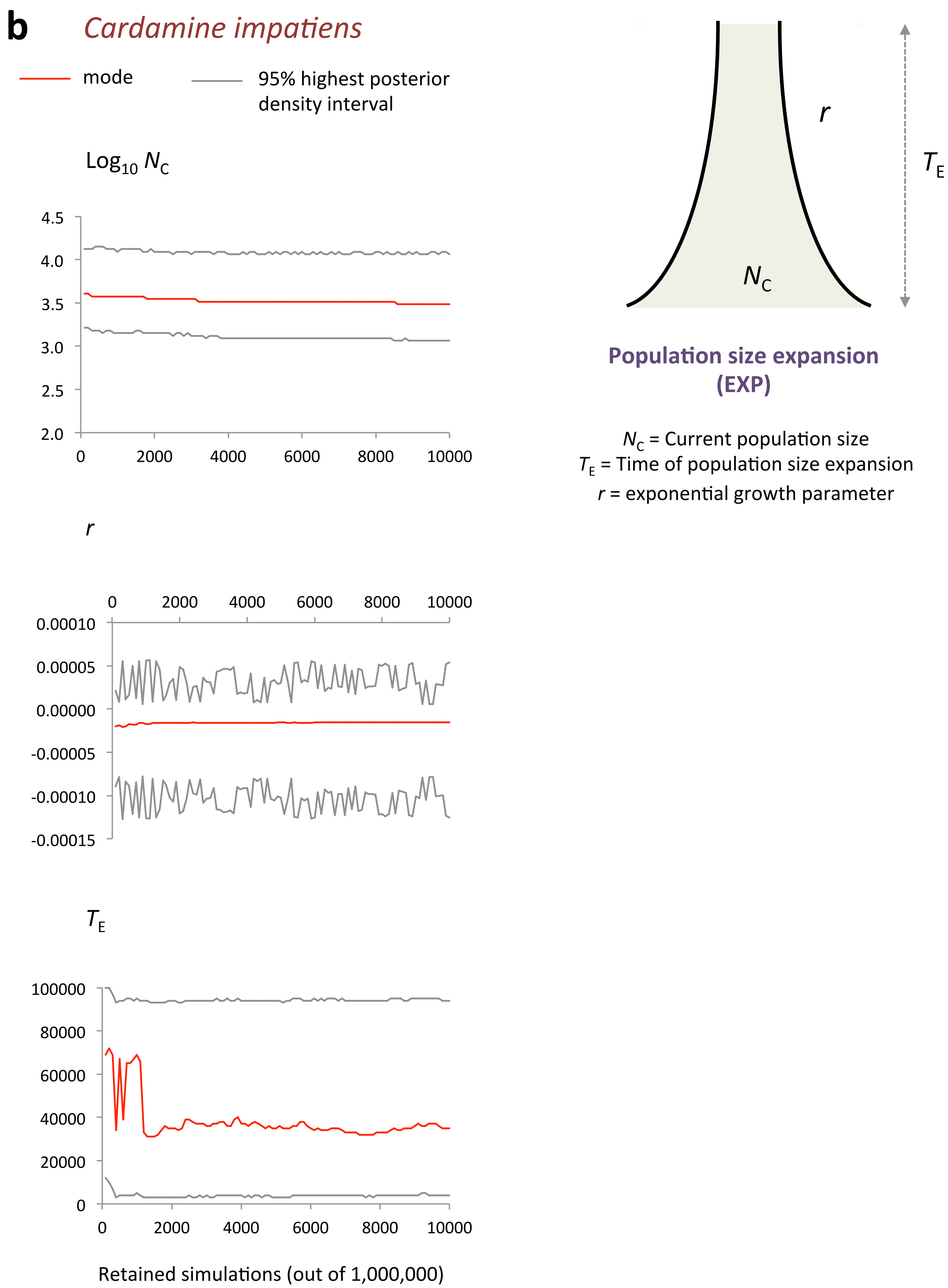
b
Cardamine impatiens
mode
95% highest posterior density interval
Log10 NC
4.5
4.0
3.5
3.0
2.5
2.0
0
2000
4000
6000
8000
10000
r
0.00010
0.00005
0.00000
-0.00005
-0.00010
-0.00015
TE
100000
80000
60000
40000
20000
Retained simulations (out of 1,000,000)
NC
TE
Population size expansion (EXP)
NC = Current population size
TE = Time of population size expansion
r = exponential growth parameter

b *Cardamine resedifolia*

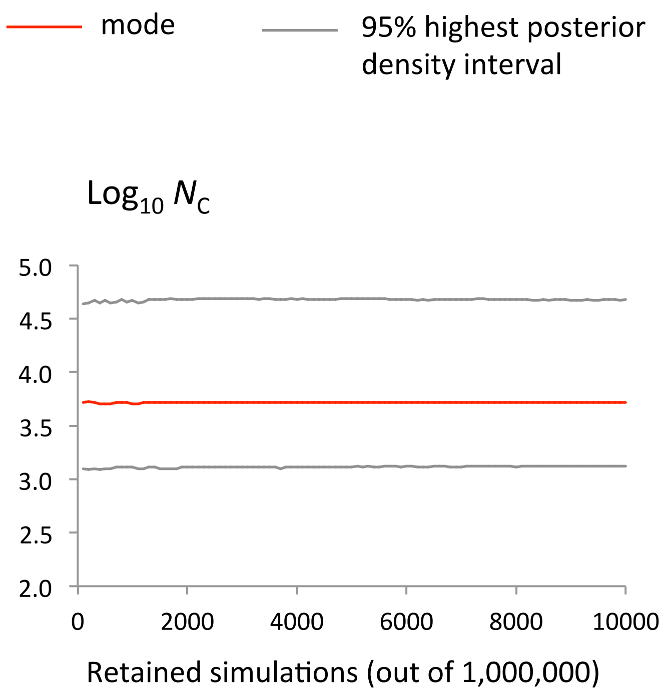

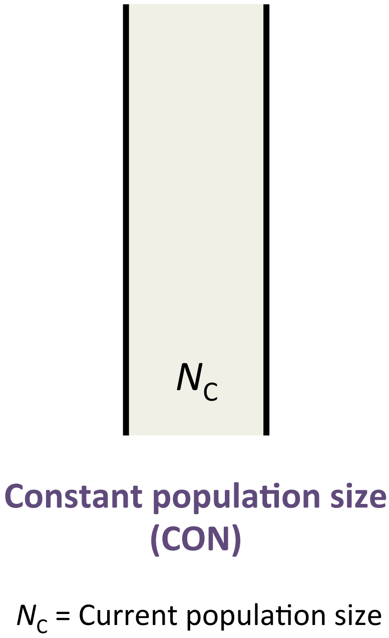

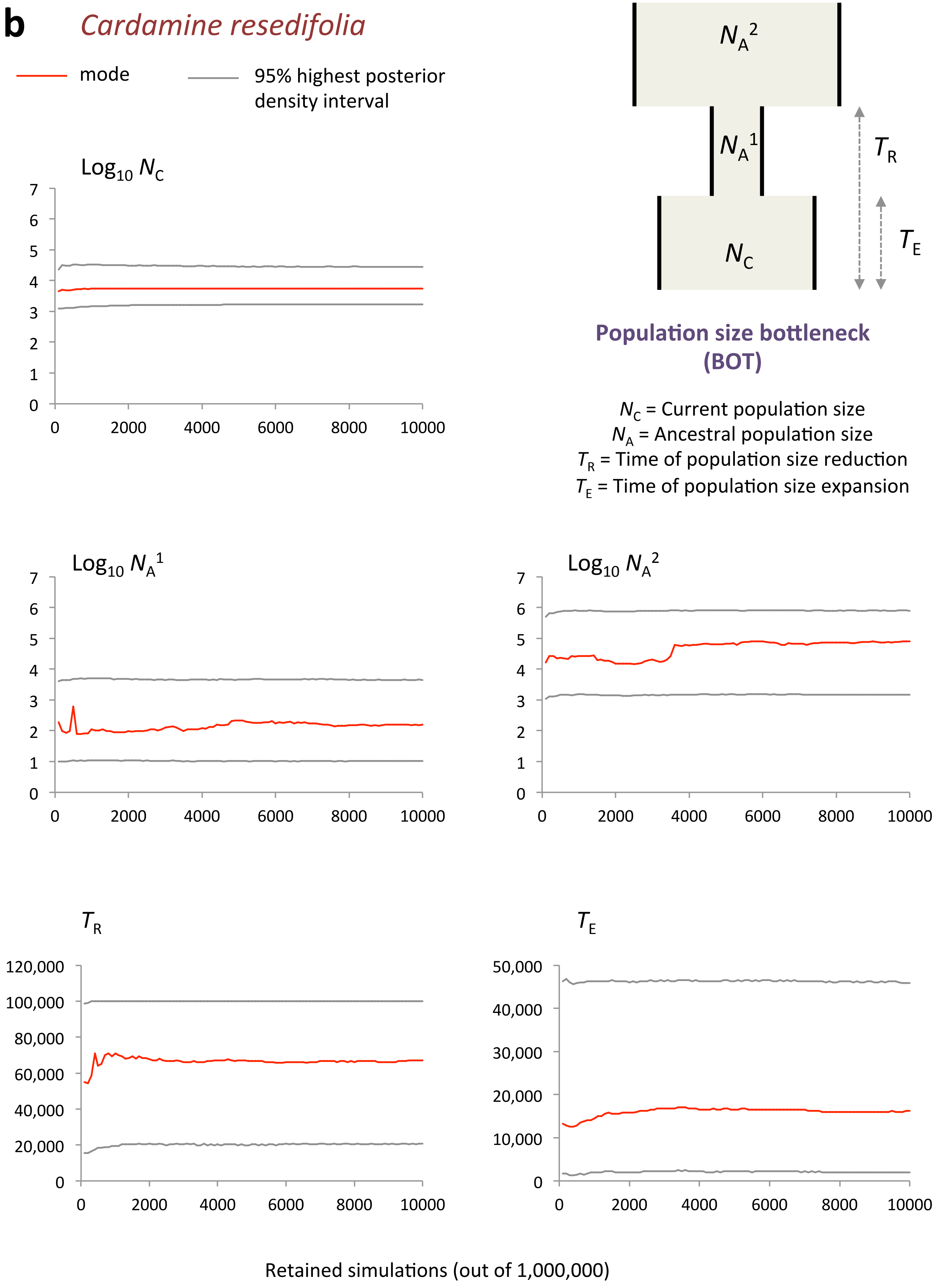
b
Cardamine resedifolia
mode
95% highest posterior density interval
Log10 NC
NA2
NA1
NC
TR
TE
Population size bottleneck (BOT)
NC = Current population size
NA = Ancestral population size
TR = Time of population size reduction
TE = Time of population size expansion
Log10 NA1
Log10 NA2
TR
TE
Retained simulations (out of 1,000,000)

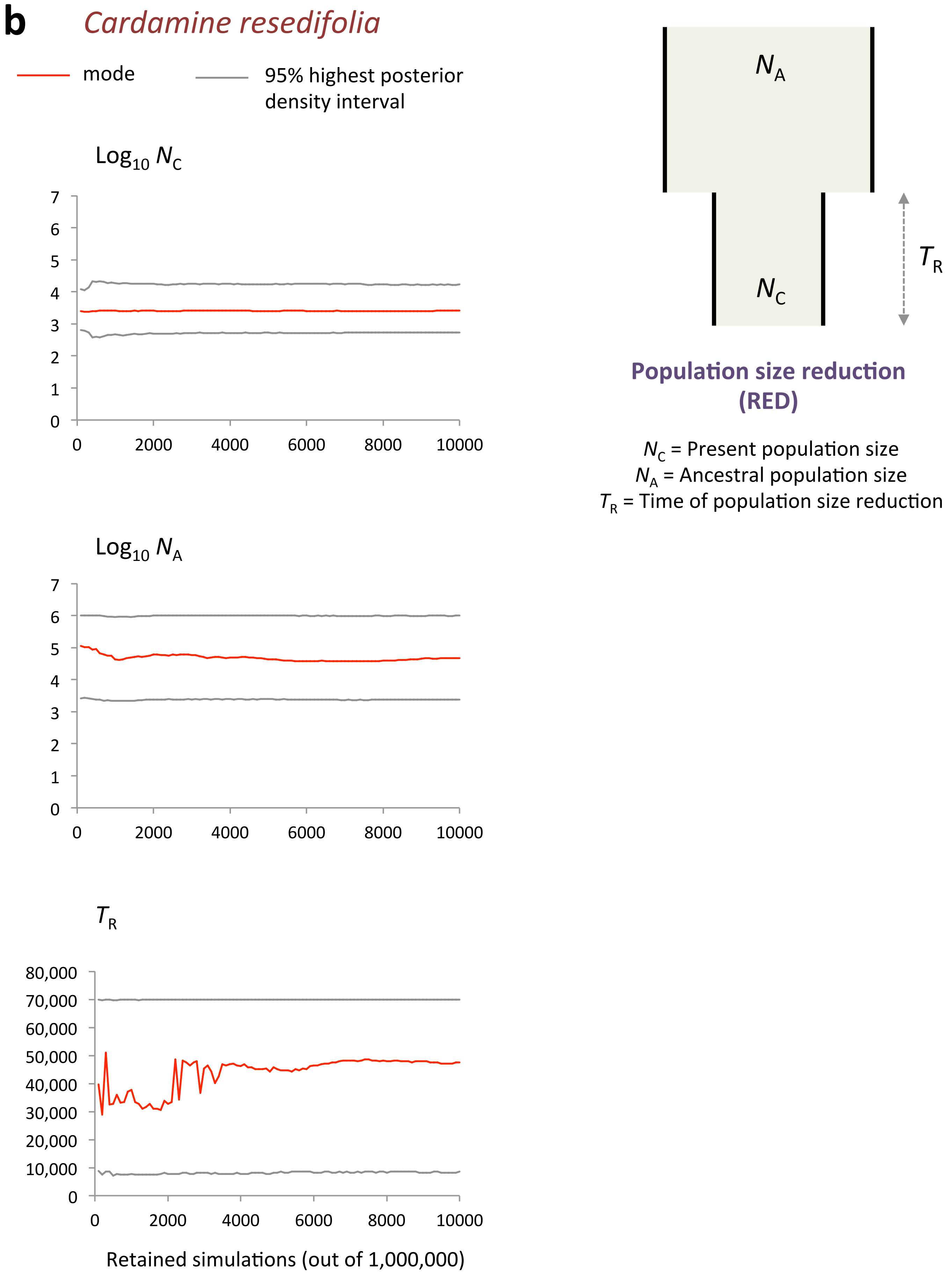
b
Cardamine resedifolia
mode
95% highest posterior density interval
Log10 NC
0
1
2
3
4
5
6
7
0
2000
4000
6000
8000
10000
Log10 NA
0
1
2
3
4
5
6
7
0
2000
4000
6000
8000
10000
TR
0
10,000
20,000
30,000
40,000
50,000
60,000
70,000
80,000
0
2000
4000
6000
8000
10000
Retained simulations (out of 1,000,000)
NA
NC
TR
Population size reduction (RED)
NC = Present population size
NA = Ancestral population size
TR = Time of population size reduction

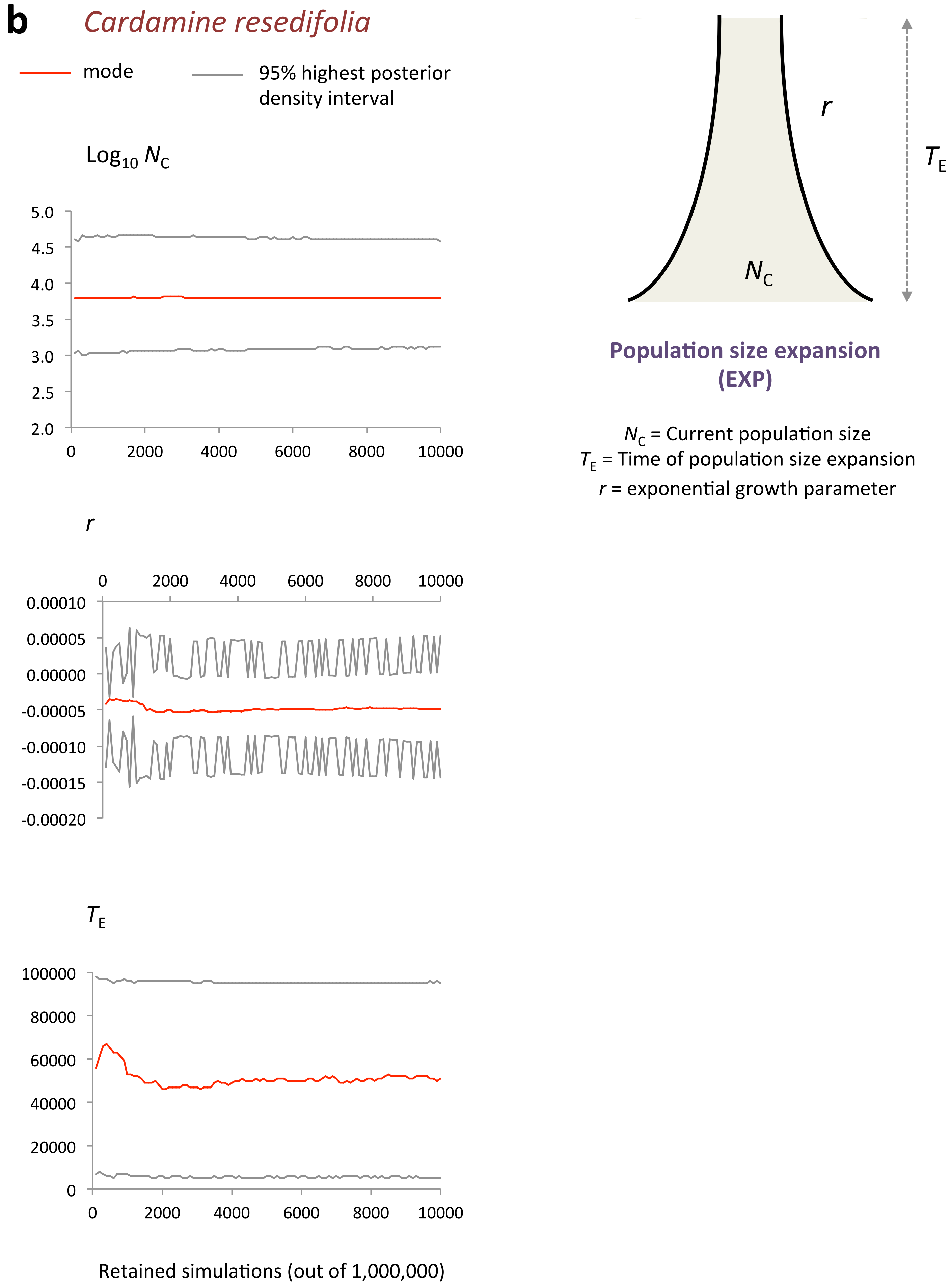
b
Cardamine resedifolia
mode
95% highest posterior density interval
Log10 NC
5.0
4.5
4.0
3.5
3.0
2.5
2.0
0
2000
4000
6000
8000
10000
r
0.00010
0.00005
0.00000
-0.00005
-0.00010
-0.00015
-0.00020
TE
100000
80000
60000
40000
20000
Retained simulations (out of 1,000,000)
r
TE
NC
Population size expansion (EXP)
NC = Current population size
TE = Time of population size expansion
r = exponential growth parameter

c

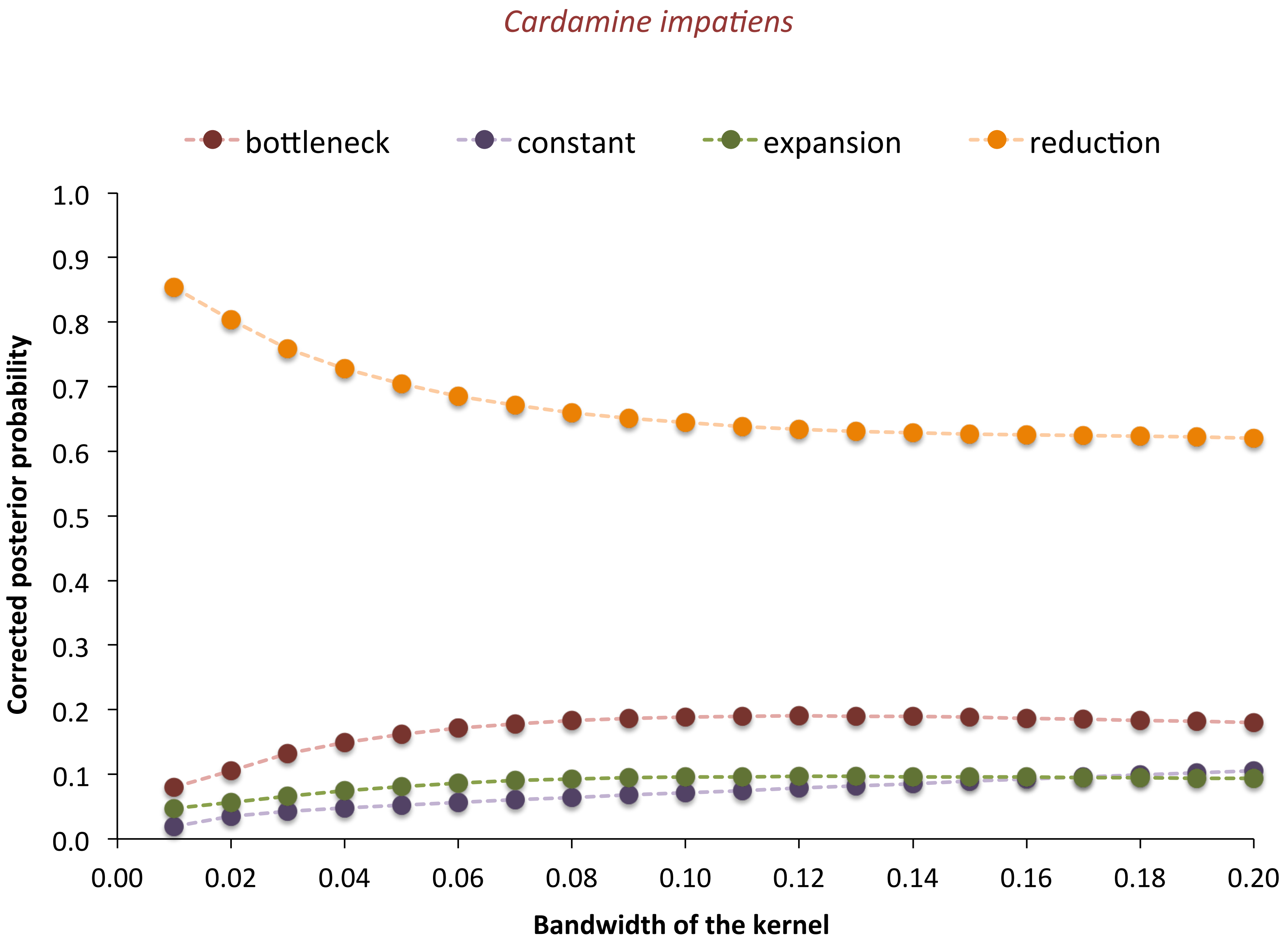
Cardamine impatiens
bottleneck
constant
expansion
reduction
Corrected posterior probability
1.0
0.9
0.8
0.7
0.6
0.5
0.4
0.3
0.2
0.1
0.0
0.00
0.02
0.04
0.06
0.08
0.10
0.12
0.14
0.16
0.18
0.20
Bandwidth of the kernel

c

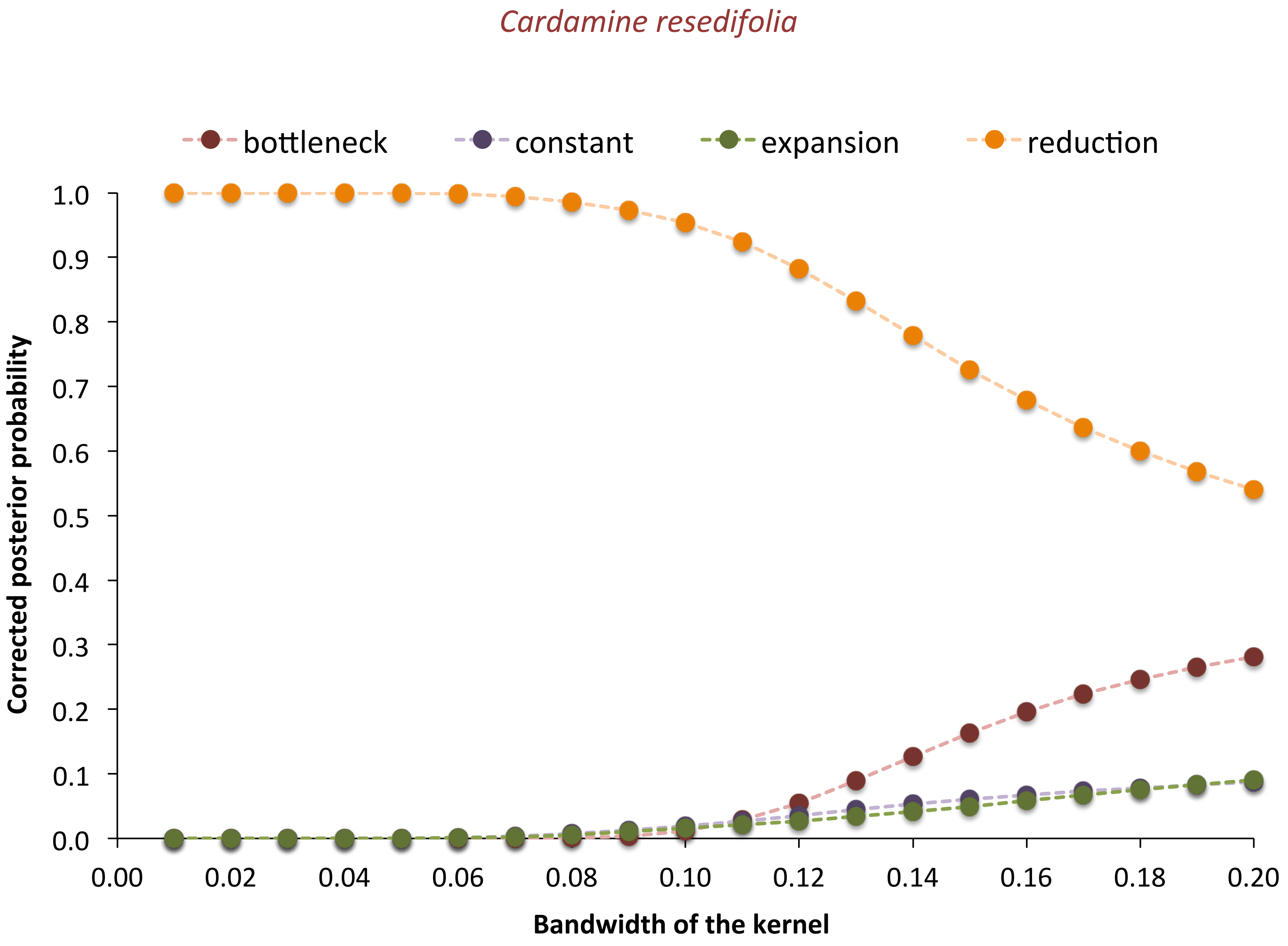

Cardamine resedifolia
bottleneck
constant
expansion
reduction
Corrected posterior probability
1.0
0.9
0.8
0.7
0.6
0.5
0.4
0.3
0.2
0.1
0.0
0.00
0.02
0.04
0.06
0.08
0.10
0.12
0.14
0.16
0.18
0.20
Bandwidth of the kernel

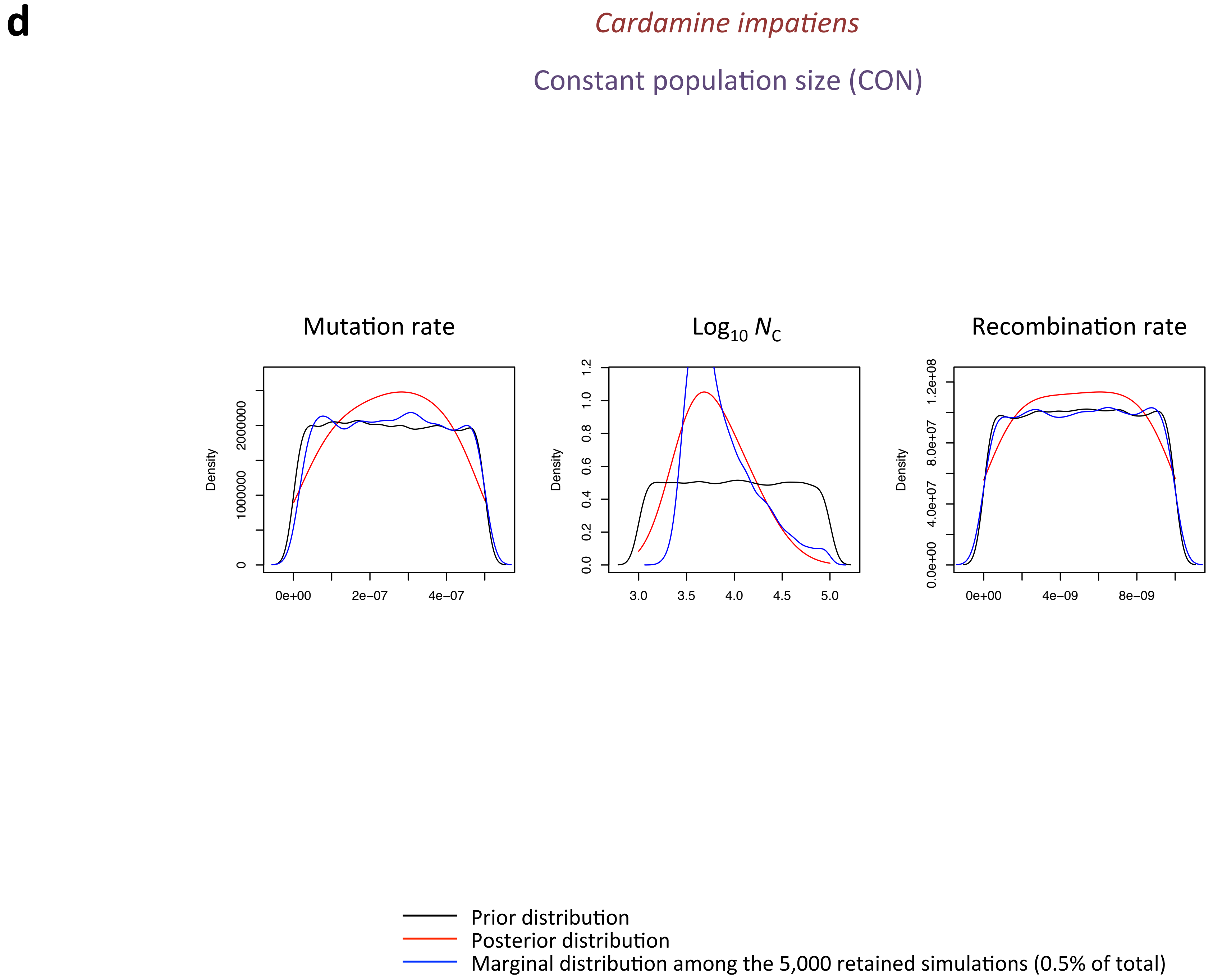
d
Cardamine impatiens
Constant population size (CON)
Mutation rate
Density
0
1000000
2000000
0e+00
2e−07
4e−07
Log10 NC
Density
0.0
0.2
0.4
0.6
0.8
1.0
1.2
3.0
3.5
4.0
4.5
5.0
Recombination rate
Density
0.0e+00
4.0e+07
8.0e+07
1.2e+08
0e+00
4e−09
8e−09
Prior distribution
Posterior distribution
Marginal distribution among the 5,000 retained simulations (0.5% of total)

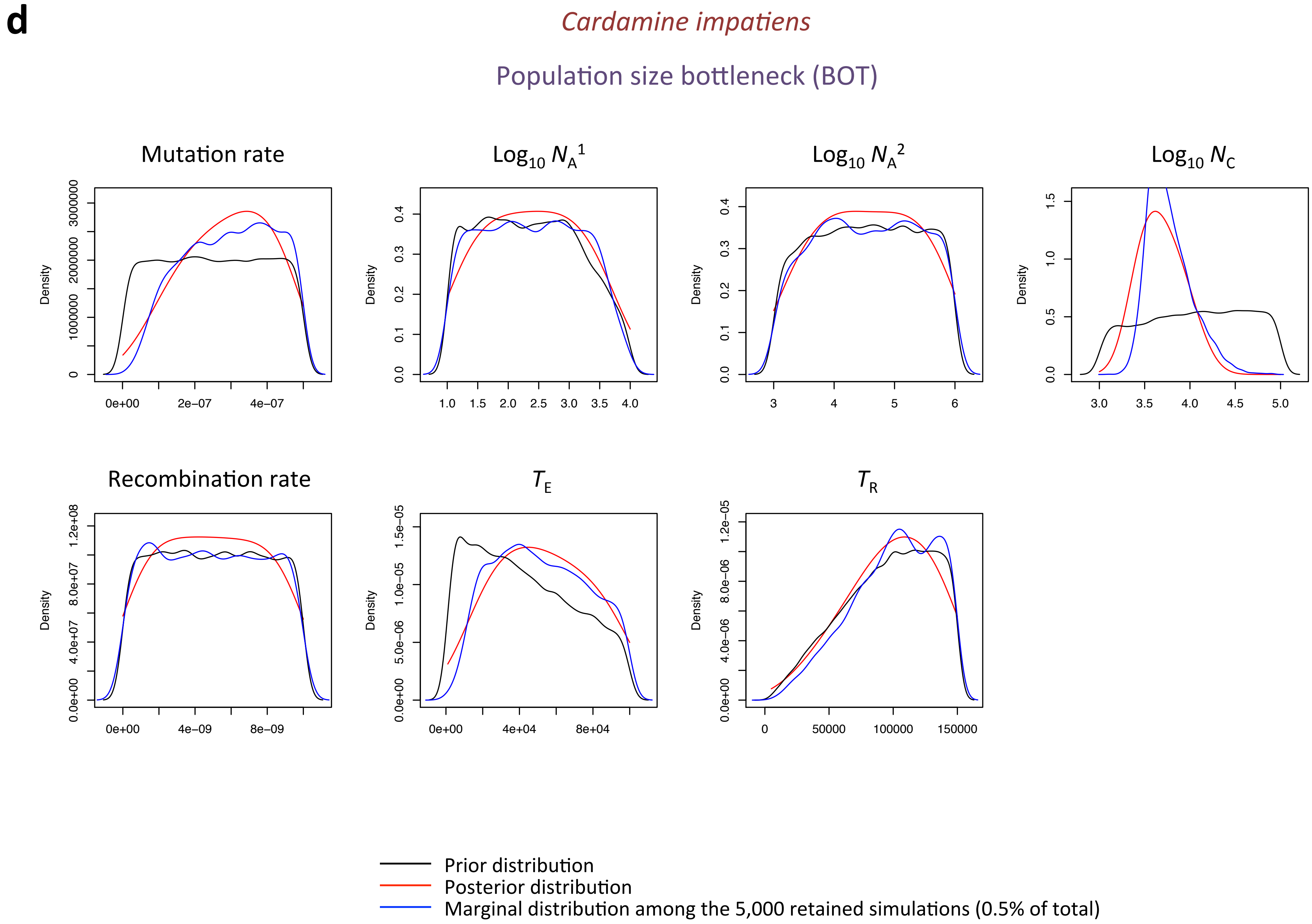
d
Cardamine impatiens
Population size bottleneck (BOT)
Mutation rate
Log10 NA1
Log10 NA2
Log10 NC
Recombination rate
TE
TR
Density
Prior distribution
Posterior distribution
Marginal distribution among the 5,000 retained simulations (0.5% of total)

d

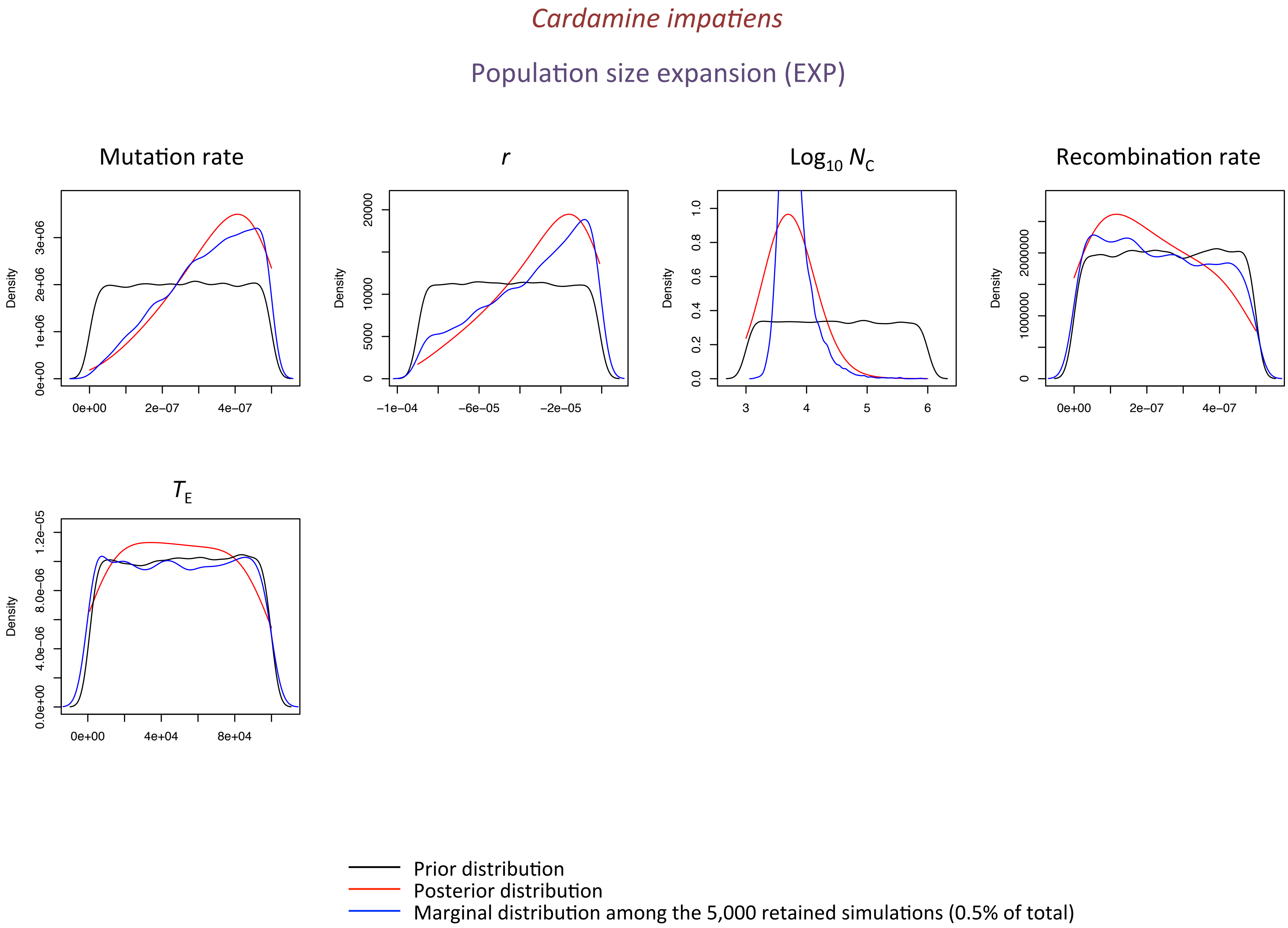

Cardamine impatiens
Population size expansion (EXP)
Mutation rate
r
Log10 *N*C
Recombination rate
*T*E
Density
Prior distribution
Posterior distribution
Marginal distribution among the 5,000 retained simulations (0.5% of total)

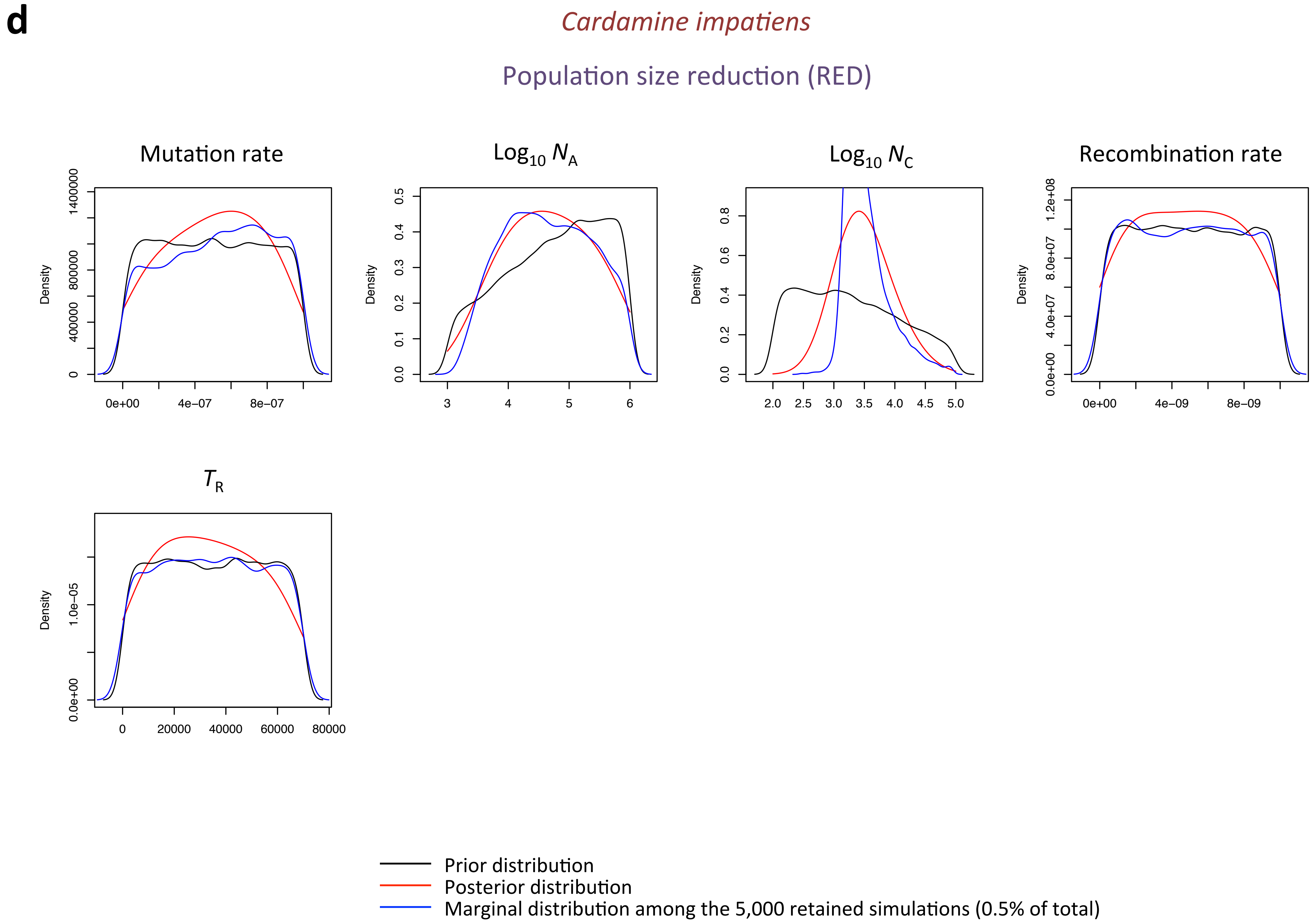
d
Cardamine impatiens
Population size reduction (RED)
Mutation rate
Log10 NA
Log10 NC
Recombination rate
TR
Density
Prior distribution
Posterior distribution
Marginal distribution among the 5,000 retained simulations (0.5% of total)

d

Cardamine resedifolia

Constant population size (CON)

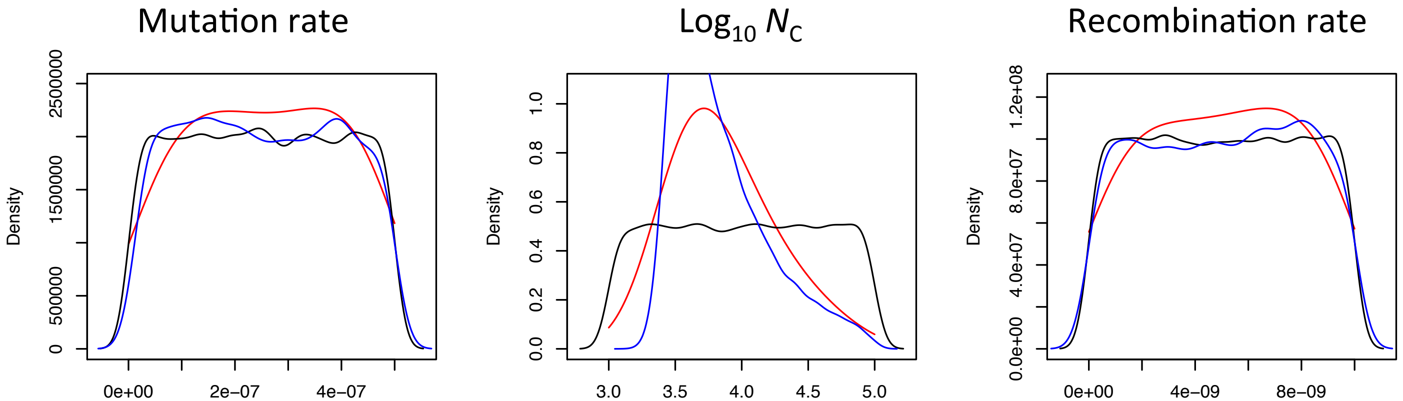

Prior distribution
Posterior distribution
Marginal distribution among the 5,000 retained simulations (0.5% of total)

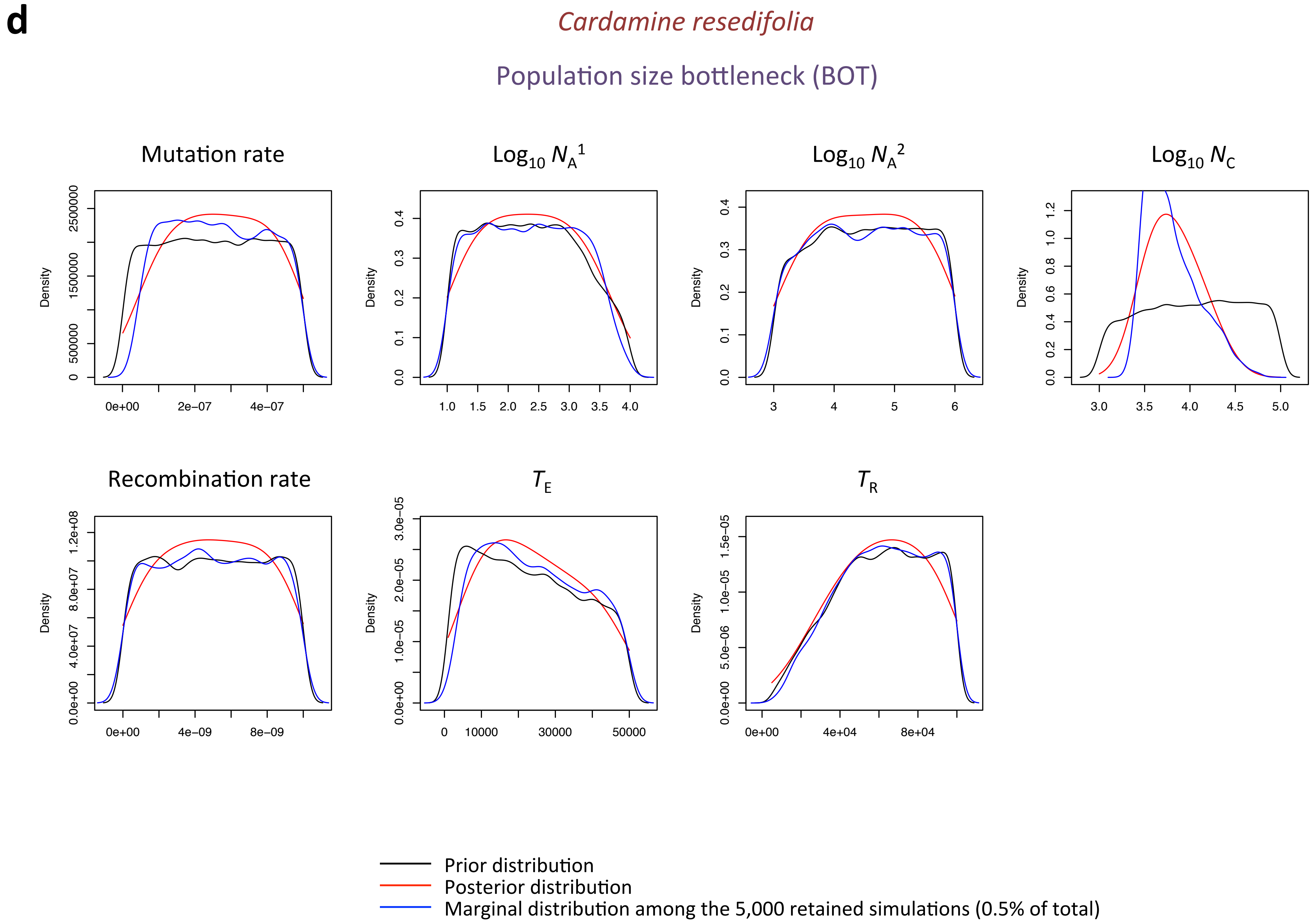
d
Cardamine resedifolia
Population size bottleneck (BOT)
Mutation rate
Log10 NA1
Log10 NA2
Log10 NC
Recombination rate
TE
TR
Density
Prior distribution
Posterior distribution
Marginal distribution among the 5,000 retained simulations (0.5% of total)

d

Cardamine resedifolia

Population size expansion (EXP)

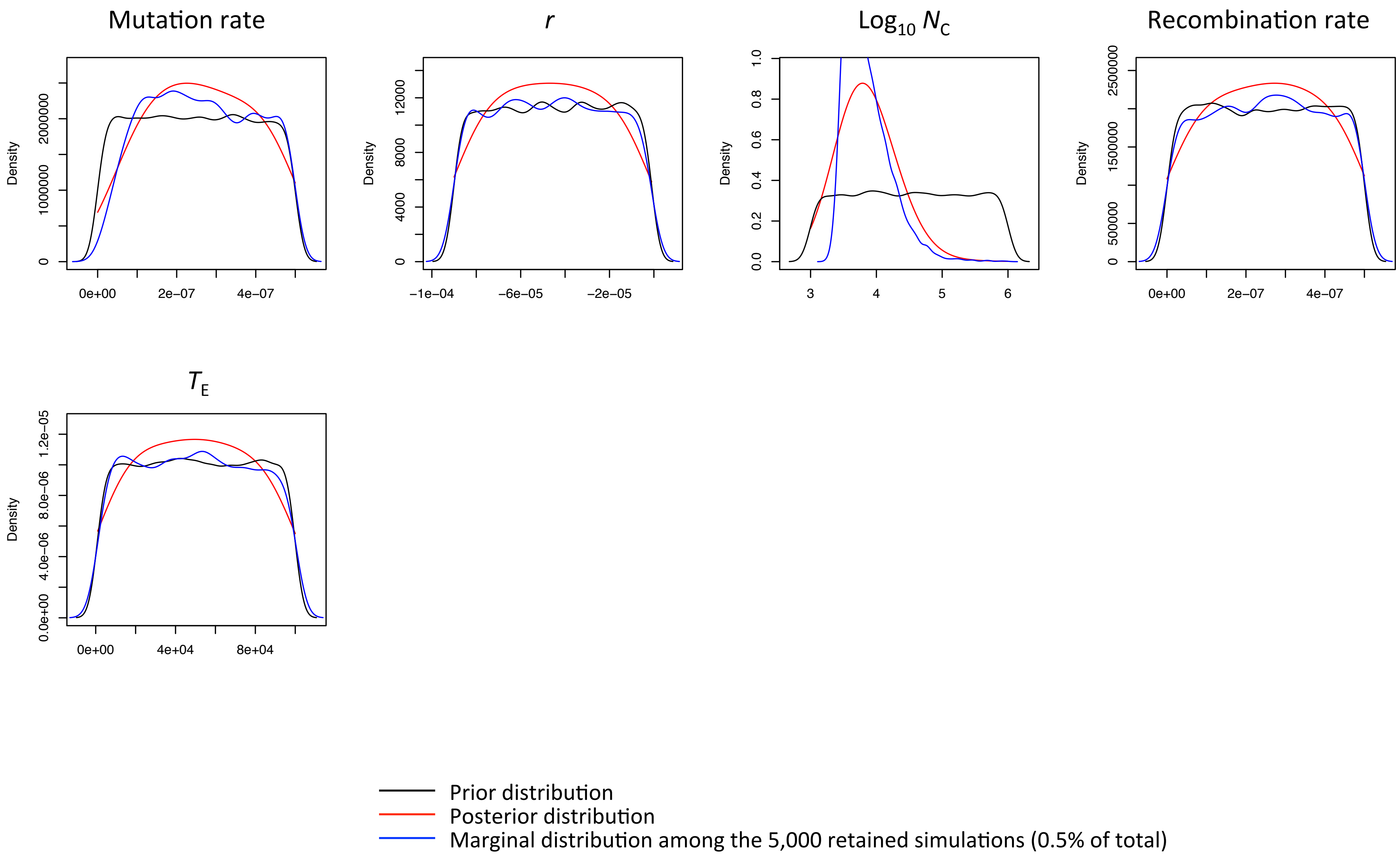

d

Cardamine resedifolia

Population size reduction (RED)

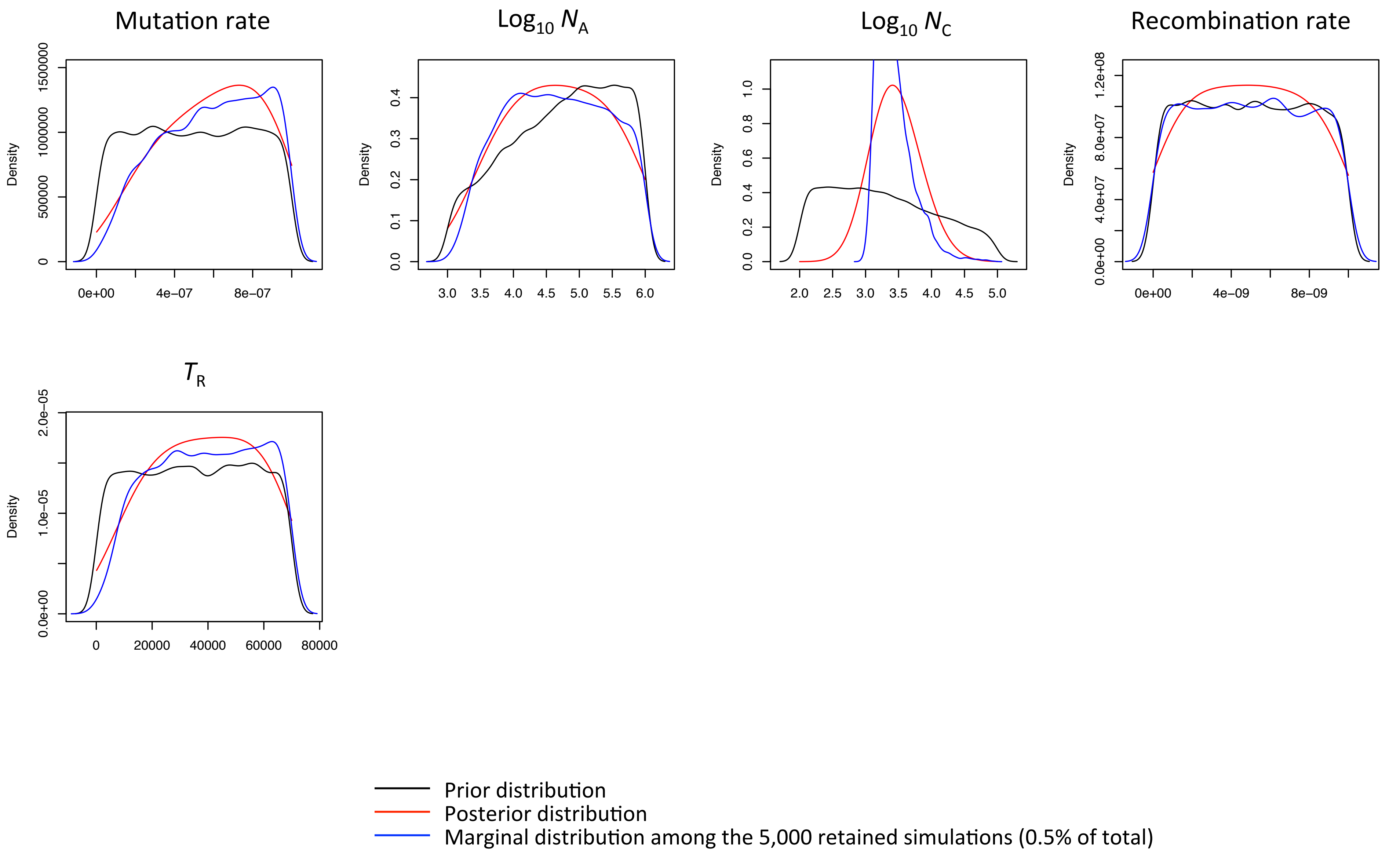

Supplement: S1 Fig — For each demographic model we report the marginal densities (a) and modes (b), the bandwidth of the kernel (c), and the posterior probabilities of each of the model parameters (d). (PDF) [file pone.0125199.s001.pdf]
