# Supplementary material for: Demographic History, Population Structure, and Local Adaptation in Alpine Populations of Cardamine impatiens and Cardamine resedifolia
Source: PLoS One. 2015 May 1;10(5):e0125199. doi: 10.1371/journal.pone.0125199 (PMC4416911; doi:10.1371/journal.pone.0125199)
Supplement: S1 Table — (PDF) [file pone.0125199.s002.pdf]

Table S1. Sampling localities.

Pop ID [a]	Locality	Coordinates [b]		Altitude [b]	Alt. class [c]	Mean rainfall (mm)		Mean temperature (°C)	
						Spring	Summer	Spring	Summer
C. impatiens									
Boa	Lago di Malga Boazzo	46.009415N	10.511593E	1276	High	247.2	268.3	8.9	15.9
Dao	Daone	45.944384N	10.627532E	548	Low	255.9	244.8	13.7	19.1
Bor	Valle di Borzago	46.103404N	10.678468E	1261	High	247.2	268.3	9.3	16.0
Cad	Caderzone	46.132648N	10.760835E	717	Low	215.7	235.3	12.6	18.4
Gag	Loc. Gaggia, Andalo	46.147058N	11.001796E	1431	High	212.8	224.1	9.8	16.2
Spo	Spormaggiore	46.230983N	11.057449E	455	Low	212.8	224.1	15.0	20.3
Vca	Val Campelle	46.139752N	11.506643E	1247	High	250.9	275.8	10.2	15.8
Vsu	Agnedo, Valsugana	46.044452N	11.508479E	337	Low	250.9	275.8	16.5	21.5
Imr	Imer	46.151236N	11.784693E	749	Low	276.3	287.5	13.4	18.4
Sma	S. Martino di Castrozza	46.257076N	11.813765E	1557	High	268.2	346.2	8.5	14.7
C. resedifolia									
Ton	North of Passo Tonale	46.288410N	10.574801E	2526	High	296.4	319.4	2.5	12.8
Sta	Stavel	46.273822N	10.659961E	1246	Low	296.4	319.4	9.1	16.2
Cor	Laghi di Cornisello	46.218817N	10.725025E	2129	High	247.2	268.3	3.6	12.8
Nam	Val Nambrone	46.216673N	10.753543E	1418	Low	215.7	235.3	7.5	15.3
Ter	Passo Termen	46.483397N	10.973414E	2274	High	199.6	248.3	4.5	12.9

Rum	Rumo	46.456403N	10.988123E	1427	Low	199.6	248.3	9.0	15.5
Cal	Val Calamento	46.154864N	11.418083E	1575	Low	220.5	251.6	8.6	14.8
Crv	Cimon Rava	46.127745N	11.576776E	2187	High	250.9	275.8	5.6	13.0
Can	Canazei	46.471241N	11.762693E	1638	Low	268.2	346.2	8.2	14.7
Fed	Passo Fedaia	46.458995N	11.889919E	2191	High	268.2	346.2	4.7	12.9

[a] Population identification code.

[b] Geographical coordinates (latitude/longitude) and altitude (in meters above sea level) refer to the central point of the sampling transect.

[c] Plants were sampled at altitudes close to either the upper (High) or lower (Low) limit of their distribution range.
