# Supplementary material for: Demographic History, Population Structure, and Local Adaptation in Alpine Populations of Cardamine impatiens and Cardamine resedifolia
Source: PLoS One. 2015 May 1;10(5):e0125199. doi: 10.1371/journal.pone.0125199 (PMC4416911; doi:10.1371/journal.pone.0125199)
Supplement: S2 Table — (PDF) [file pone.0125199.s003.pdf]

Table S2. Gene function as inferred from the *A. thaliana* orthologue.

Gene [a]	*c/n* [b]	Description
AT1G07890	*c**	Ascorbate peroxidase 1 (APX1); L-ascorbate peroxidase activity; ending in seed dormancy, response to reactive oxygen species, response to cadmium ion, response to salt stress, response to heat.
AT1G61520	*c*	LHCA3; chlorophyll binding; photosynthesis, light harvesting, photosynthesis.
AT1G63440	*nc*	HEAVY METAL ATPASE 5 (HMA5); ATPase activity, coupled to transmembrane movement of ions, phosphorylative mechanism.
AT1G69070	*nc*	Molecular function unknown.
AT1G77490	*c*	THYLAKOIDAL ASCORBATE PEROXIDASE (TAPX); L-ascorbate peroxidase activity; response to oxidative stress.
AT2G15970	*c*	COR413-PM1; molecular_function unknown; cold acclimation, cellular response to water deprivation, response to abscisic acid stimulus.
AT2G16500	*c*	ARGININE DECARBOXYLASE 1 (ADC1); arginine decarboxylase activity; response to oxidative stress, response to salt stress, response to cold, seed development.
AT2G22590	*nc*	Transferase, transferring glycosyl groups.
AT2G31610	*c**	40S ribosomal protein S3 (RPS3A); structural constituent of ribosome; response to salt stress, translation, response to abiotic stimulus.
AT2G36530	*c*	LOS2; phosphopyruvate hydratase activity, copper ion binding; response to cadmium ion, response to salt stress, response to light stimulus, response to cold, response to abscisic acid stimulus.

AT2G42540	*c*	COLD-REGULATED 15A (COR15A); molecular function unknown.
AT2G44060	*c**	Late embryogenesis abundant family protein; molecular function unknown; ending in seed dormancy, response to cadmium, response to desiccation.
AT4G23850	*c**	Long-chain-fatty-acid--CoA ligase / synthetase; fatty acid biosynthetic process.
AT4G29350	*c**	PROFILIN 2 (PFN2); protein binding; actin polymerization or depolymerization, cytoskeleton organization.
AT5G01950	*nc*	ATP binding / kinase; protein serine/threonine kinase activity; transmembrane receptor protein tyrosine kinase signaling pathway.
AT5G11490	*nc*	Adaptin family protein; protein transporter activity, protein binding: intracellular protein transport.
AT5G14420	*nc*	RING domain Ligase2 (RGLG2); ubiquitin-protein ligase activity; N-terminal protein myristoylation, cytokinin metabolic process, auxin metabolic process.
AT5G50100	*nc*	Unknown functions.
AT5G51750	*nc*	ARABIDOPSIS THALIANA SUBTILASE 1.3 (ATSBT1.3); identical protein binding, serine-type endopeptidase activity.

[a] TAIR-ID of the *A. thaliana* orthologue.
[b] *c* = candidate gene (* after Ometto *et al.* 2012); *nc* = non-candidate (neutral) gene.
