# Supplementary material for: Demographic History, Population Structure, and Local Adaptation in Alpine Populations of Cardamine impatiens and Cardamine resedifolia
Source: PLoS One. 2015 May 1;10(5):e0125199. doi: 10.1371/journal.pone.0125199 (PMC4416911; doi:10.1371/journal.pone.0125199)
Supplement: S3 Table — (PDF) [file pone.0125199.s004.pdf]

Table S3. Primers used to amplify the genes used in the study.

Amplicon [a]	Primers		Ann. T (°C) [b]	
	Forward	Reverse	*imp*	*res*
AT1G07890-a	ATGACGAAGAACTACCCAACCG	CAAGCAGACAATATCAATCAGCC	60	60
AT1G07890-b	CCTGATATTCCTTTCCACCCTG	CGTATTTCTCGACTAAAGGACGG	60	60
AT1G49750-a	ACACTTGCTTCTTGGAAAGGCG	GAACTCATAACCCACTATCACAG	60	60
AT1G49750-b	ATGACGCAGGTTCACGGGTCC	CGTTAGTGCGTTGTAAGAAACCG	60	60
AT1G61520-a	CTTGTGTCTTCTTCACTTACCTCC	GTGGAATCAAACCTGCCTTGCC	58	65
AT1G63440-a	TGCTCTTGGGCTGGCTACTCC	GTTTATGGCGACAAGAATCCCG	60	60
AT1G63440-b	GGAGTATAAGCCACCGTTAAAGG	AGCCCATAGGTAGTTGAGACGG	60	60
AT1G69070-a	TTTCAGTTGAAGAAGAGCAGCC	TAGTAATTCAAAGTTCAAGGGC	60	60
AT1G69070-b	ACCGAATCAGGATAGGAAACGC	CGGATAAATAATAAGGCTTCAGG	60	60
AT1G69070-c	ATCTCTTCTGGTCGTGATATTGCC	CACAAACCCTCTAAGAGTCTCG	60	60
AT1G77490-a	AAGGTATCAAAGTGCTTCTCCG	TCTTACTTCTTCTGGACACTGG	58	60
AT1G77490-b	CTCTTACGCCGACTTATTCCAG	CTACTGCTTCTCCAGGTCCAG	58	60
AT1G77490-c	GATAACCTGAGACAAAGTACACG	TTCTCTCATTTATGTACCTCGGG	60	60
AT2G15970-a	CGAATGGGTTGCTTCAATCGCC	GACGACGAGAGCAATGATCCCG	60	60
AT2G16500-a	CTGTTGCTTATAGTCTTGAGGAGT	AGTAACATTCCCAAATAGTATCCC	60	60
AT2G16500-b	CACTTCGATTCCTGATTTCTGGG	CATTTCTCATCCTTACCGCCGC	60	60
AT2G22590-a	TTCCAAGAGTACCGTACTTCGCC	TCGATGATCGTTCCCCAACCG	60	60
AT2G22590-b	AAGTTTGAAGATACCGACACG	CTATCCATGTCTCCAAACACTCC	60	60

AT2G31610-a	TTCAGAAGAGATTCAAGTTTCCTC	GACGAAACCATGTATCCATCC	60	60
AT2G31610-b	ATAGTTAAGGTTTGGACTAGGT	GAGCACCTGGAGCAACAACCTG	60	60
AT2G36530-a	ACAGACGTTGGTGATGAAGGTGG	TGCTGACCAACAAATCATCACCG	60	60
AT2G36530-b	AGTACCGAATTGTGTCCATTGAGG	ACGGGTTTGCGGAAGTTGGCTC	60	60
AT2G42540-a	GCTGGAAGACTGAGTTCGTCGT	TAGCCGTCACATATTCCGAAGC	60	60
AT2G44060-a	AGTGACTTTGATCTATGATGAC	ATCATCATCTTCCTTCTTCAGACG	60	60
AT4G23850-a	TGGATACTACAAACGTGAAGACC	AATGGATTCCTTTGCCTTTGCG	60	60
AT4G23850-b	GTGTGGGTGTACGGGAACAGC	TTTGTGGTCTTGTACATTTCATCG	60	60
AT4G29350-a	TCAGCCACCTCACTTCCGCC	CACCTAGAAATAGCCCTGTTGG	58	60
AT4G29350-b	GATTGTGGTTTAGTTGAAGCCTGA	CAGACTCAATAAGGTAATCGCCG	60	60
AT5G01950-a	AGGTTATGGAAAGGTTTACAGAGG	TTAGGCACATCCTCTTCGTCC	60	60
AT5G01950-b	CTCTACCGTCACACAGAAGCC	GCACTGACGAAGACGACACCG	60	57
AT5G11490-a	GTTCGGTTCCATCTCTTAACTGC	GAAGTAGGTCCTTGTCATTTGCC	60	60
AT5G14420-a	ACAATACAAGGCGACAATAGAGC	GAGCTTTAATCTGGTCTGAATCGG	60	60
AT5G14420-b	AGCGAATTTGACGATAATATCCCG	AATGCCATATCTTTCGGGTTGCTC	60	60
AT5G50100-a	AATATCAAGTTAGAGCAATACAAGG	TCCAACTTCTTCATATAATCTCC	60	55
AT5G50100-b	TCGTCAGAAGAGAATCAAGGGC	CACGAATAGCTTCTAAAGATGGC	60	60
AT5G51750-a	AAGATCGTGATATGTGACCGTGG	GTGCAGGACTTAAATCTTGAGTGC	56	60

[a] Amplicons were named after the TAIR-ID of the *A. thaliana* orthologous genes.
[b] Annealing temperature used for PCR amplification of *C. impatiens* (*imp*) and *C. resedifolia* (*res*) genomic DNA.
