# Supplementary material for: Demographic History, Population Structure, and Local Adaptation in Alpine Populations of Cardamine impatiens and Cardamine resedifolia
Source: PLoS One. 2015 May 1;10(5):e0125199. doi: 10.1371/journal.pone.0125199 (PMC4416911; doi:10.1371/journal.pone.0125199)
Supplement: S4 Table — (PDF) [file pone.0125199.s005.pdf]

Table S4. Polymorphism in the coding regions of *C. impatiens*.

Gene [a]	n [b]	Ex. [c]	Synonymous sites					Non-synonymous sites				
			L_{eff} [d]	P_S [e]	π [f]	θ_W [f]	F_S [g]	L_{eff} [d]	P_{NS} [e]	π [f]	θ_W [f]	F_{NS} [g]
Cimp-AT1G07890	116	6	133	0	0.00000	0.00000	27	452	1	0.00004	0.00042	21
Cimp-AT1G61520	120	3	82	0	0.00000	0.00000	11	239	2	0.00014	0.00156	8
Cimp-AT1G63440	120	4	211	5	0.00474	0.00443	32	668	4	0.00155	0.00112	14
Cimp-AT1G69070	110	6	247	4	0.00164	0.00307	32	905	10	0.00105	0.00210	46
Cimp-AT1G77490	116	9	169	1	0.00010	0.00111	37	560	2	0.00069	0.00067	12
Cimp-AT2G15970	120	3	92	0	0.00000	0.00000	25	280	3	0.00491	0.00200	11
Cimp-AT2G16500	120	1	243	10	0.00455	0.00768	52	774	4	0.00075	0.00096	25
Cimp-AT2G22590	112	1	187	3	0.00254	0.00303	61	641	3	0.00222	0.00088	40
Cimp-AT2G31610	120	4	102	4	0.01339	0.00729	25	321	4	0.00026	0.00233	14
Cimp-AT2G36530	114	7	146	5	0.00405	0.00646	28	502	0	0.00000	0.00000	14
Cimp-AT2G42540	118	2	46	2	0.00073	0.00808	12	161	2	0.00324	0.00233	14
Cimp-AT2G44060	118	1	112	2	0.00288	0.00334	31	392	3	0.00013	0.00143	8
Cimp-AT4G23850	120	4	104	0	0.00000	0.00000	23	376	0	0.00000	0.00000	13
Cimp-AT4G29350	116	3	69	2	0.00050	0.00547	17	219	1	0.00008	0.00086	9
Cimp-AT5G01950	120	4	187	0	0.00000	0.00000	33	614	1	0.00003	0.00030	18
Cimp-AT5G11490	120	3	83	5	0.00605	0.01118	7	283	0	0.00000	0.00000	3
Cimp-AT5G14420	120	5	106	2	0.00077	0.00353	16	347	1	0.00081	0.00054	15
Cimp-AT5G50100	120	5	88	7	0.01724	0.01490	31	311	3	0.00142	0.00180	12

Cimp-AT5G51750	114	1	165	11	0.00196	0.01255	44	498	11	0.00117	0.00416	13
All genes [l]	117.6 (3.2)	72	2,572	63	0.00322 (0.00471)	0.00485 (0.00453)	544	8,543	55	0.00097 (0.00130)	0.00123 (0.00105)	310

[a] Name refers to the TAIR-ID of the *A. thaliana* orthologue (ATnGnnnnn).

[b] Number of sequenced haplotypes.

[c] Number of (partial) exons.

[d] Number of effective sites (missing data and sites with gaps are excluded).

[e] Levels of polymorphic sites.

[f] Levels of nucleotide diversity, estimated using π and θ_W.

[g] Number of substitutions between *C. resedifolia* and *A. thaliana*.

[h] Probability of the McDonald-Kreitman test.
