# Supplementary material for: Demographic History, Population Structure, and Local Adaptation in Alpine Populations of Cardamine impatiens and Cardamine resedifolia
Source: PLoS One. 2015 May 1;10(5):e0125199. doi: 10.1371/journal.pone.0125199 (PMC4416911; doi:10.1371/journal.pone.0125199)
Supplement: S5 Table — (PDF) [file pone.0125199.s006.pdf]

Table S5. Polymorphism in the coding regions of *C. resedifolia*.

Gene [a]	n [b]	Ex. [c]	Synonymous sites					Non-synonymous sites				
			L_{eff} [d]	P_S [e]	π [f]	θ_W [f]	F_S [g]	L_{eff} [d]	P_{NS} [e]	π [f]	θ_W [f]	F_{NS} [g]
Cres-AT1G07890	114	6	135	1	0.00086	0.00139	28	450	1	0.00060	0.00042	20
Cres-AT1G61520	120	3	82	4	0.00616	0.00916	15	240	2	0.00027	0.00156	7
Cres-AT1G63440	116	4	211	11	0.01017	0.00979	34	665	7	0.00088	0.00198	23
Cres-AT1G69070	104	6	240	8	0.00849	0.00640	34	861	15	0.00204	0.00334	42
Cres-AT1G77490	110	9	171	4	0.00074	0.00444	37	561	4	0.00157	0.00135	12
Cres-AT2G15970	120	3	94	4	0.00362	0.00797	33	278	4	0.00042	0.00268	9
Cres-AT2G16500	112	1	241	3	0.00348	0.00235	48	773	8	0.00211	0.00196	31
Cres-AT2G22590	118	1	183	3	0.00295	0.00306	60	636	4	0.00021	0.00118	48
Cres-AT2G31610	118	4	101	0	0.00000	0.00000	20	319	2	0.00016	0.00117	12
Cres-AT2G36530	120	7	144	8	0.00351	0.01035	25	501	3	0.00010	0.00112	15
Cres-AT2G42540	120	2	45	0	0.00000	0.00000	13	154	0	0.00000	0.00000	15
Cres-AT2G44060	120	1	111	0	0.00000	0.00000	27	393	1	0.00013	0.00047	8
Cres-AT4G23850	120	4	103	3	0.00541	0.00541	29	377	3	0.00151	0.00149	13
Cres-AT4G29350	112	3	70	2	0.00675	0.00543	19	224	3	0.00032	0.00253	9
Cres-AT5G01950	120	4	186	4	0.00845	0.00400	31	615	0	0.00000	0.00000	17
Cres-AT5G11490	120	3	88	1	0.00074	0.00212	7	293	1	0.00033	0.00064	3
Cres-AT5G14420	120	5	106	0	0.00000	0.00000	17	341	2	0.00015	0.00110	20
Cres-AT5G50100	114	5	89	0	0.00000	0.00000	27	311	2	0.00146	0.00121	12

Cres-AT5G51750	114	1	162	9	0.00371	0.01050	44	495	9	0.00052	0.00342	13
All genes [1]	116.4 (4.5)	72	2,561	65	0.00342 (0.00332)	0.00434 (0.00380)	548	8,485	71	0.00067 (0.00070)	0.00145 (0.00100)	329

[a] Name refers to the TAIR-ID of the *A. thaliana* orthologue (ATnGnnnnn).

[b] Number of sequenced haplotypes.

[c] Number of (partial) exons.

[d] Number of effective sites (missing data and sites with gaps are excluded).

[e] Levels of polymorphic sites.

[f] Levels of nucleotide diversity, estimated using π and θ_W.

[g] Number of substitutions between *C. resedifolia* and *A. thaliana*.

[h] Probability of the McDonald-Kreitman test.
